# Uncovering the relationship between trace element exposure, cognitive function, and dietary inflammation index in elderly americans from the National Health and Nutrition Examination Survey 2011–2014

**DOI:** 10.1186/s12889-024-20060-4

**Published:** 2024-09-16

**Authors:** Chunlan Tang, Min Shen, Hang Hong

**Affiliations:** 1 Department of Ophthalmology, The Affiliated People’s Hospital of Ningbo University, Ningbo, Zhejiang 315040 China; 2 School of Public Health, Health Science Center, Ningbo University, Ningbo, Zhejiang 315211 China; 3 Reference Laboratory, Medical System Biotechnology Co., Ltd. Ningbo, Zhejiang, 315104 China

**Keywords:** Dietary inflammatory index, Trace elements, Cognitive function, NHANES

## Abstract

**Background:**

The consequences of trace element exposure on cognitive function in elderly adults have been recognized as primarily attributed to the inflammatory response. It is noteworthy that diet can either exacerbate or reduce the inflammatory response. Despite this, there have been limited studies about the effects of diet on the relationship between trace element exposure and cognitive function.

**Methods:**

A cross-sectional study utilized data from the 2011–2014 NHANES survey to explore the association between trace element exposure and cognitive function in elderly adults. The study enrolled 1726 participants, and generalized linear regression model (GLM), Bayesian kernel machine regression model (BKMR), weighted quantile sum regression (WQS), and quantile g-computation regression analysis (Qg-comp) were conducted to assess the impact of five trace elements (lead, cadmium, mercury, manganese, and selenium) in blood on cognitive function under the anti-inflammatory and pro-inflammatory diet.

**Results:**

The GLM analysis showed a positive correlation between selenium (Se) and both the instant recall test (IRT) and digit symbol substitution test (DSST) (β = 2.06, 95% CI: 0.70 ~ 3.41; and β = 6.41, 95% CI: 2.35 ~ 10.46, respectively). In contrast, cadmium (Cd) was negatively associated with DSST (β = -1.17, 95% CI: -2.13~ -0.22), and lead (Pb) was negatively associated with IRT (β = -0.47, 95% CI: -0.82~ -0.11). For the animal fluency test (AFT), the highest quartile of manganese (Mn) was negatively associated with the lowest quartile (β = -0.72, 95% CI: -1.34~-0.10), while mercury (Hg) showed no significant associations with cognitive function tests. Subgroup analysis revealed the effects of Cd on IRT and DSST and Se on DSST under the pro-inflammatory diet. Furthermore, The BKMR analysis showed an inverted U-shaped curve with the negative impact of trace element mixtures and DSST and a linearly negative trend with IRT in the pro-inflammatory diet. Among them, Cd was emphasized as the most potent risk factor, and Se was the most vital protective factor for IRT and DSST in WQS and Qg-comp analysis.

**Conclusions:**

The study suggests that a high-quality diet might alleviate the adverse effects of Cd on IRT and DSST. High Se levels were also associated with better IRT and DSST scores in the pro-inflammatory diet. These findings provide valuable insights into the connection between diet, trace element exposure, and cognitive function in elderly adults.

**Supplementary Information:**

The online version contains supplementary material available at 10.1186/s12889-024-20060-4.

## Background

Global life expectancy has given rise to a significant global public health concern regarding the cognitive health of elderly adults. With the number of older adults experiencing cognitive impairment or dementia on the rise, it is estimated that this population will reach 78 million by 2030 and a staggering 139 million by 2050 [1]. A decline in various mental abilities, including memory loss, poor concentration, impaired information processing, confusion or memory loss, and semantic comprehension errors, characterizes cognitive impairment. It can also lead to abnormal reasoning and decision-making processes [2, 3]. This decline can profoundly impact the daily activities of elderly adults, ultimately reducing their quality of life. Numerous studies indicate that cognitive decline is closely associated with physical, psychological, social, and lifestyle risk factors [4–6].

Furthermore, several investigations have demonstrated that exposure to some trace element pollution, especially heavy metals, can impact cognitive function in older adults [7, 8]. Most heavy metals occur naturally, but a few are derived from anthropogenic sources. Heavy metals released into the environment can enter the human body through the food chain, posing potential threats to human health [9]. The precise mechanisms by which this occurs remain unclear; however, trace elements are known to trigger neuroinflammation [10, 11], which is a complex process involving various factors such as direct toxic effects, immune response, oxidative stress, and neuronal apoptosis. These processes interact and promote the development of neuroinflammation. Trace element ions can bind to neuronal proteins, causing neuronal damage, neuroinflammation, and nervous system dysfunction [12, 13].

Additionally, they can stimulate the immune system to produce an inflammatory response, resulting in neuroinflammation [14, 15]. Trace element exposure promotes the production of reactive oxygen species (ROS), which can attack cell components and cause cell damage and inflammation [16, 17]. They can also induce neuronal apoptosis, exacerbating neuroinflammation and damaging the nervous system [18]. However, there is ongoing debate surrounding the potential impacts of some trace elements. While some studies have reported adverse cognitive effects associated with manganese (Mn) exposure [19, 20], a dietary survey conducted on a substantial Chinese population in 2023 revealed that Mn did not demonstrate any significant associations with cognitive function [21].

The constituents of daily food intake are complex, necessitating an exploration of specific dietary conditions to understand their impact on inflammation and cognitive impairment. The Dietary Inflammatory Index (DII) is a recognized indicator of overall dietary inflammation, calculated by merging diverse food components. Research has demonstrated that a higher DII score positively correlates with cognitive impairment [22, 23]. However, limited studies have evaluated whether dietary intake of antioxidants and anti-inflammatory compounds can modify the effect of trace element exposure on markers of neuroinflammation. It is hypothesized that an anti-inflammatory or pro-inflammatory diet may attenuate or enhance inflammation and subsequently reduce or increase trace elements-induced neurotoxicity.

The present study explores whether the association between blood trace elements and cognitive function is modified by overall diet quality measured by the DII score. The study will employ three statistical models, namely the Bayesian kernel machine regression (BKMR) model, weighted quantile sum (WQS) model, and quantile g-computation (Qg-comp) model, to analyze data from the National Health and Nutrition Examination Survey (NHANES). The primary objective is to investigate whether the overall quality of an individual’s diet can strengthen or weaken the relationship between blood trace elements and cognitive function. By understanding the complex interplay between trace element exposures, dietary inflammatory potential, and cognitive health, this study aims to provide valuable insights for developing targeted interventions and public health strategies to promote healthy aging and prevent cognitive decline in older adults.

## Methods

### Study population

**Fig. 1:**
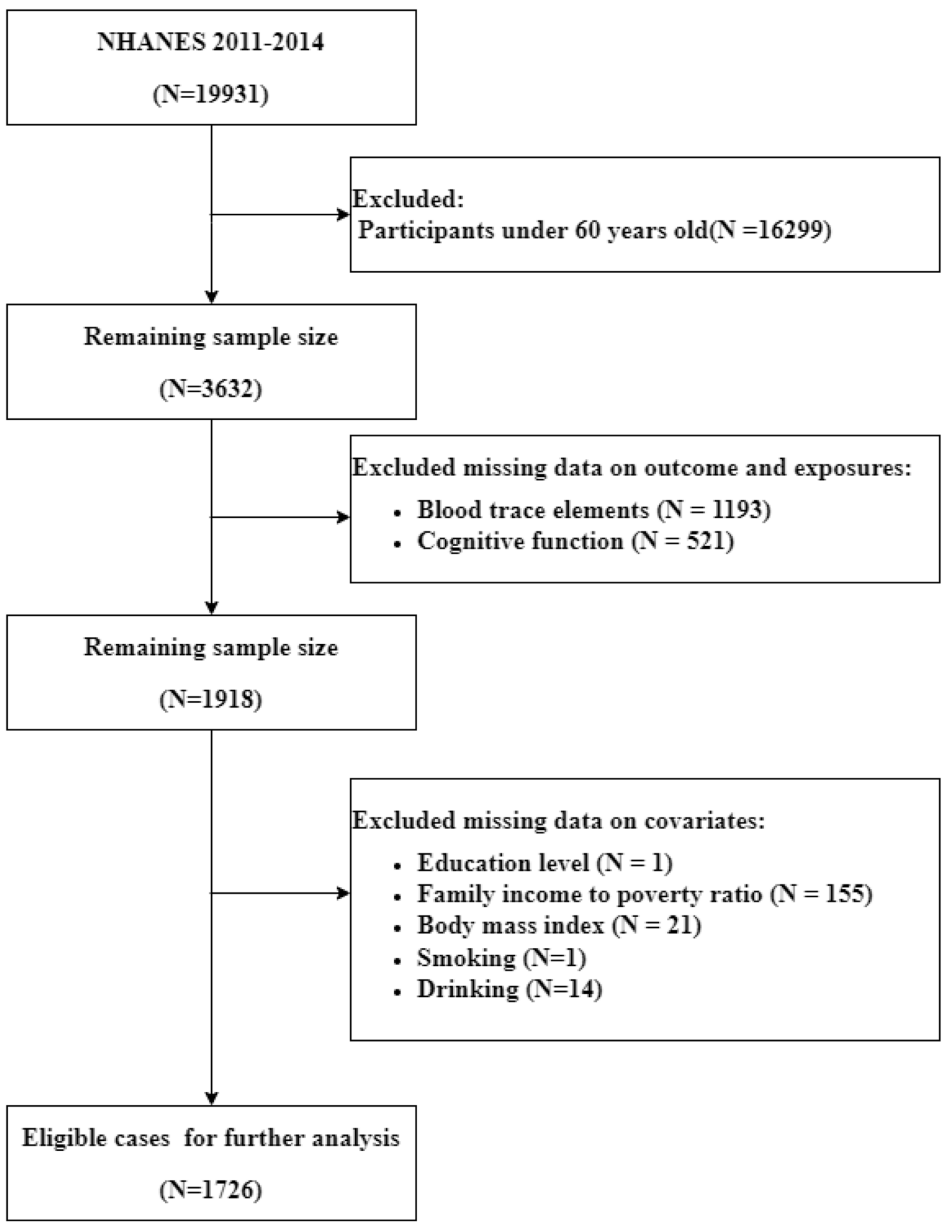
Flow chart of the selection of eligible subjects

The present study primarily analyzed data from two cycles (2011–2012 and 2013–2014) of NHANES, a cross-sectional survey conducted by the Centers for Disease Control and Prevention (CDC) to evaluate the health and nutrition status of the non-institutionalized population in the US. The study received approval from the National Center for Health Statistics (NCHS), and all participants provided their informed consent. Out of 19,931 respondents, data were collected from 3,632 participants aged 60 or older. Participants with incomplete information on blood trace element levels, cognitive tests, and covariates were excluded from the analysis. Ultimately, the study comprised 1,726 individuals aged 60 or older (Fig. 1).

### Measurement

#### Cognitive function

The cognitive abilities of the individuals were assessed using four tests: the Instant Recall Test (IRT), the Delayed Recall Test (DRT), the Animal Fluency Test (AFT), and the Digit Symbol Substitution Test (DSST). IRT and DRT were used to evaluate the acquisition of new linguistic material immediately and after a certain period of delay. The AFT was used to assess executive function, while the DSST was used to determine the participants’ cognitive abilities, such as reaction time, sustained attention, and working memory. All test scores were standardized to ensure consistency with the composite score. The resulting scores serve as a measure of cognitive function, with higher scores indicating superior performance.

#### Trace elements

The CDC’s Division of Laboratory Sciences at the National Center for Environmental Health processed, stored, and analyzed whole blood specimens for trace element concentrations. Specifically, the attention of lead (Pb), cadmium (Cd), total mercury (Hg), Mn, and selenium (Se) were analyzed using inductively coupled plasma mass spectrometry (ICP-MS). In cases where the blood trace element levels fall below the lower limit of detection (LLOD), they were replaced by the result of dividing the limit by the square root of 2 (LLOD/sqrt [2]). The blood’s concentration of all trace elements was transformed using a natural logarithm to obtain nearly normal distributions.

#### Dietary inflammatory index

The Dietary Inflammatory Index (DII) was derived from data obtained through two 24-hour dietary recall interviews [24]. Calculating a Z-score involves subtracting the global per capita daily intake from the daily intake of a specific nutrient. This result was then divided by the standard deviation of the global per capita daily intake. The resulting Z-score was converted into a percentage, which fell from 0 to 1. This percentage was doubled and subtracted by 1, resulting in a symmetrical distribution centered at “0”. Finally, the total inflammation score was obtained by multiplying the percentage received for each dietary component by the total inflammation score. This calculation was done for twenty-seven nutrients, including alcohol, β-carotene, caffeine, carbohydrate, cholesterol, folic acid, fiber, iron, magnesium, n-3 fatty acids, n-6 fatty acids, total monounsaturated fatty acids, niacin, total polyunsaturated fatty acids, protein, total saturated fatty acids, Se, total fat, riboflavin, thiamin, vitamin A, B12, B6, C, D, E, and zinc. Participants were categorized into DII ≥ 0 and DII < 0, signifying pro-inflammatory and anti-inflammatory diets, respectively.

### Covariate assessment

The study incorporated several demographic covariates in the analysis, specifically sex, age (60–69, 70–79, or ≥ 80), race (American, Mexican other Hispanic, non-Hispanic white, non-Hispanic black, or other), marital status (never married, married/living with partner, or widowed/divorced/separated), family income to poverty ratio (≤ 1.3, 1.3–3.5, or > 3.5), drinking status (having at least 12 alcohol drinks per year or not), body mass index (BMI) (< 18.5 kg/cm², ≥ 18.5 to < 25 kg/cm², ≥ 25 to < 30 kg/cm², or ≥ 30 kg/cm²), smoking status (having at least 100 cigarettes in their lifetime or not), and education level (less than 9th grade, 9-11th grade, high school grad/GED, some college or AA degree, or college graduate or above). Hyperlipidemia was diagnosed when a person had either low-density cholesterol levels of 160 mg/dL or higher, total cholesterol levels of 240 mg/dL or higher, or lipid-lowering medication. Hypertension was diagnosed by a physician or a systolic blood pressure of ≥ 130 mmHg, a diastolic blood pressure of ≥ 80 mmHg, or current use of hypertension medications. Individuals with diabetes were identified based on any of the following criteria: (**A**) a hemoglobin A1C concentration of 6.5% or higher or a fasting plasma glucose level of 126 mg/dL or higher; (**B**) individuals who answered positively to the question: “Has a doctor told you that you have diabetes?” or “Are you currently taking insulin?” Physical activity was measured using a unit called Metabolic Equivalent of Task (MET) scores. The scores were assigned as follows: 8 for vigorous work-related exercise, 4 for moderate work-related activity, 4 for walking or bicycling, 8 for vigorous leisure-time physical activity, and 4 for moderate leisure-time physical activity. Weekly minutes were added and multiplied by each activity’s MET score to determine the total amount of physical activity. The US physical activity guidelines defined a high level of physical activity as ≥ 600 MET*min/week and a low level of physical activity as < 600 MET*min/week.

### Statistical analysis

The categorical variables of four cognitive test scores were presented as medians, accompanied by their respective interquartile ranges (IQR). The Kruskal-Wallis test was employed for comparison. The correlation between the five trace elements was evaluated using Pearson correlation coefficients. Cognitive function scores were analyzed for association with trace element exposure using generalized linear regression models (GLM). Furthermore, we used quartiles of trace element exposure and a trend test across increasing exposure groups in multivariable models, then rerunning the related regression model. Subgroup analysis was conducted to determine any variations in the correlation between trace element exposure and cognitive function, stratified by the DII group (anti-inflammatory and pro-inflammatory diets), using stratified linear regression models. Likelihood-ratio tests inspected the modifications and interactions of subgroups.

The potential interactions and collective impacts of trace element exposure on cognitive function were analyzed using the BKMR statistical modeling. The researchers investigated the nonlinear relationship between exposure and outcome by employing exposure-response cross-sections for a single independent variable and outcome. The study further controlled for other variables by keeping them constant at the median. Bivariate exposure-response profiles can be used to represent the interaction between different mixture compositions. This interaction can be understood as the potential impact of one chemical’s slope curve at various modifications of another chemical (25th, 50th, and 75th) while the remaining variables are held constant at the median. The association plot offered a comprehensive analysis of the impact of the mixture on the outcome. It displayed how the outcome was expected to change based on different percentiles of exposure variables compared to when they were in the media. The iteration was performed using the Markov Chain Monte Carlo method with a fixed value of 30,000.

The WQS regression model is a statistical approach that uses mixed-effects strategies to analyze the relationship between multiple chemical exposures and outcomes. It evaluates this relationship by computing a weighted index and determining the proportional impact of each exposure. The weights assigned to each exposure are between 0 and 1, and the sum of all weights equals 1. The potential association between trace element exposure and cognitive function was investigated by the WQS model while controlling for all relevant covariates. The present study involved randomly partitioning original data into a training set (40%) and a validation set (60%) to derive weight values for each trace element exposure. Subsequently, the bootstrap method with a sample size of 3000 was applied to the data to infer the statistical properties of the derived weight values.

We used the Qg-comp model to address the WQS regression model’s limitations in determining the association’s direction. Qg-comp model provides a reliable means of predicting the impact of exposure mixtures. However, in large samples where the assumptions of WQS regression hold, Qg-comp can also yield equivalent estimates. The qgcomp.noboot function was applied to establish a linear model for cognitive function. We used positive or negative weighting indices for each trace element in the blood and subsequently segmented each into quartiles to determine its overall effect.

We developed five models in sensitivity analysis to examine the association between various factors and the outcome. Model 1 had no confounding variables, while Model 2 adjusted for sex, age, race, education level, income, and marital status. Model 3 added adjustments for physical activity, drinking, smoking habits, BMI, and DII. Model 4 accounted for diabetes, hypertension, and hyperlipidemia. Lastly, Model 5 adjusted for four other trace elements. All statistical analyses were performed using the R software (version 4.3.2) and packages “survey”, “bkmr”, “gWQS”, and “Qgcomp”. A significance level of *P* < 0.05 was considered.

## Results

### Descriptive statistics

The study comprised 1726 participants, of which 853 were male and 873 were female. Sociodemographic data, such as sex, age, ethnicity, education level, income, marital status, drinking and smoking habits, BMI, and physical activity, was collected and subsequently categorized as presented in Table 1. The results indicated that women who were non-Hispanic Whites, highly educated, and had high incomes had higher scores on cognitive function tests. Furthermore, poor cognitive performance was more prevalent in adults with diabetes and hypertension than in those with normal cognitive performance. Notably, poor cognitive performance in the pro-inflammatory diet was observed compared to that in the anti-inflammatory diet. The study also revealed that older adults with high blood Cd and Pd levels had low cognitive function scores on IRT, DRT, AFT, and DDST. Conversely, high blood Hg and Se levels presented high cognitive function scores. Figure S1 presents the correlation matrix for the five trace elements analyzed in this study. Cd and Pb exhibited a moderate positive correlation (*r* = 0.27, *p* < 0.001). Mn showed a weak positive correlation with Cd (*r* = 0.15, *p* < 0.001) and Hg (*r* = 0.10, *p* < 0.001). Hg showed a weak positive correlation with Se (*r* = 0.16, *p* < 0.001). Se had a weak negative correlation with Cd (*r* = -0.07, *p* < 0.001) and Pb (*r* = -0.01, *p* < 0.001). Table S1 provides trace element concentrations in the study population.

**Table 1:** Demographic characteristics of study participants

Variables	Total number (n, %)	IRT (median, IQR)	DRT (median, IQR)	AFT (median, IQR)	DSST (median, IQR)
Total (*n* = 1726)		19.0 (16.0, 22.0)	6.0 (4.0, 8.0)	16.0 (13.0, 20.0)	46.0 (34.0, 59.0)
Sex					
female	873 (50.6)	20.0 (17.0, 23.0)	7.0 (5.0, 8.0)	16.0 (13.0, 20.0)	49.0 (36.0, 62.0)
male	853 (49.4)	18.0 (15.0, 21.0)	6.0 (4.0, 7.0)	16.0 (13.0, 20.0)	43.0 (32.0, 54.0)
*P*		< 0.001	< 0.001	0.549	< 0.001
Age group					
60–69 years	885 (51.3)	20.0 (17.0, 23.0)	7.0 (5.0, 8.0)	17.0 (14.0, 21.0)	51.0 (37.0, 63.0)
70–79 years	477 (27.6)	19.0 (16.0, 22.0)	6.0 (4.0, 8.0)	16.0 (12.0, 20.0)	44.0 (32.0, 55.0)
80 + years	364 (21.1)	17.0 (14.0, 20.0)	5.0 (3.0, 6.0)	15.0 (12.0, 18.0)	40.0 (30.0, 50.0)
*P*		< 0.001	< 0.001	< 0.001	< 0.001
Race					
Mexican American	134 (7.8)	18.0 (15.0, 21.0)	6.0 (4.0, 7.0)	16.0 (13.0, 19.0)	38.0 (26.0, 53.0)
Non-Hispanic Black	403 (23.3)	19.0 (16.0, 22.0)	6.0 (4.0, 8.0)	15.0 (11.0, 18.0)	39.0 (29.0, 52.0)
Non-Hispanic White	874 (50.6)	19.0 (16.0, 22.0)	6.0 (4.0, 8.0)	18.0 (14.0, 22.0)	50.0 (39.2, 63.0)
Other Hispanic	174 (10.1)	18.0 (15.0, 21.0)	6.0 (4.0, 7.0)	15.0 (12.0, 18.8)	35.0 (25.0, 49.0)
Other/multiracial	141 (8.2)	20.0 (16.0, 23.0)	7.0 (5.0, 8.0)	15.0 (12.0, 18.0)	50.0 (42.0, 63.0)
*P*		< 0.001	< 0.001	< 0.001	< 0.001
Education attainment					
Less Than 9th Grade	191 (11.1)	16.0 (14.0, 19.0)	5.0 (3.0, 6.0)	14.0 (11.0, 17.0)	25.0 (20.0, 33.0)
9-11th Grade	216 (12.5)	18.0 (15.0, 22.0)	6.0 (4.0, 7.0)	15.0 (11.0, 18.0)	37.0 (29.0, 46.0)
High School Grad/GED	411 (23.8)	19.0 (16.0, 21.5)	6.0 (4.0, 7.0)	16.0 (12.0, 19.0)	45.0 (34.0, 54.0)
Some College or AA degree	503 (29.1)	19.0 (16.0, 21.5)	6.0 (4.0, 7.0)	16.0 (12.0, 19.0)	45.0 (34.0, 54.0)
College Graduate or above	405 (23.5)	21.0 (17.0, 23.0)	7.0 (5.0, 8.0)	19.0 (15.0, 22.0)	56.0 (45.0, 67.0)
*P*		< 0.001	< 0.001	< 0.001	< 0.001
Income status					
<= 1.30	498 (28.9)	18.0 (15.0, 21.0)	6.0 (4.0, 7.0)	15.0 (12.0, 18.0)	36.0 (25.0, 48.0)
1.31 to < 3.5	672 (38.9)	19.0 (16.0, 22.0)	6.0 (4.0, 7.2)	16.0 (13.0, 20.0)	45.0 (34.0, 57.0)
>3.5	556 (32.2)	20.0 (17.0, 23.0)	7.0 (5.0, 8.0)	18.0 (14.0, 22.0)	55.0 (45.0, 67.0)
*P*		< 0.001	< 0.001	< 0.001	< 0.001
Marital status					
Never Married	101 (5.9)	20.0 (16.0, 23.0)	7.0 (5.0, 8.0)	15.0 (13.0, 20.0)	47.0 (35.0, 59.0)
Married/Living with partner	1010 (58.5)	19.0 (16.0, 22.0)	6.0 (4.0, 8.0)	17.0 (13.0, 21.0)	48.0 (36.0, 60.0)
Widowed/Divorced/ Separated	615 (35.6)	19.0 (16.0, 22.0)	6.0 (4.0, 7.0)	16.0 (12.0, 19.0)	43.0 (30.0, 57.0)
*P*		0.21	0.056	0.002	< 0.001
Drinking					
Non-drinker	521 (30.2)	19.0 (15.0, 22.0)	6.0 (4.0, 8.0)	15.0 (12.0, 19.0)	44.0 (31.0, 55.0)
drinker	1205 (69.8)	19.0 (16.0, 22.0)	6.0 (4.0, 8.0)	17.0 (13.0, 21.0)	48.0 (35.0, 60.0)
*P*		0.496	0.989	< 0.001	< 0.001
Smoke					
Never smoker	851 (49.3)	19.0 (16.0, 22.0)	6.0 (4.0, 8.0)	16.0 (13.0, 20.0)	48.0 (34.0, 60.5)
smoker	875 (50.7)	19.0 (16.0, 22.0)	6.0 (4.0, 7.0)	16.0 (13.0, 20.0)	45.0 (33.0, 57.0)
*P*		0.115	0.145	0.583	0.005
BMI					
Underweight(< 18.5)	23 (1.3)	21.0 (11.5, 23.0)	5.0 (3.0, 7.5)	14.0 (12.0, 19.0)	37.0 (29.5, 51.0)
Normal(18.5 to < 25)	435 (25.2)	19.0 (16.0, 22.0)	6.0 (4.0, 8.0)	16.0 (13.0, 20.0)	
Overweight(25 to < 30)	591 (34.2)	19.0 (16.0, 22.0)	6.0 (4.0, 7.0)	16.0 (13.0, 20.0)	46.0 (34.0, 60.0)
Obese(30 or greater)	677 (39.2)	19.0 (16.0, 22.0)	6.0 (5.0, 8.0)	16.0 (13.0, 20.0)	47.0 (34.0, 59.0)
*P*		0.564	0.042	0.615	0.368
Physical activity					
Low	866 (50.2)	19.0 (15.0, 22.0)	6.0 (4.0, 7.0)	15.0 (12.0, 19.0)	43.0 (31.0, 55.0)
High	860 (49.8)	19.0 (17.0, 22.0)	6.0 (5.0, 8.0)	17.0 (14.0, 21.0)	49.5 (37.0, 62.0)
*P*		< 0.001	< 0.001	< 0.001	< 0.001
Diabetes					
No	1224 (70.9)	19.0 (16.0, 22.0)	6.0 (4.0, 8.0)	17.0 (13.0, 21.0)	48.5 (36.0, 61.0)
Yes	502 (29.1)	19.0 (15.0, 22.0)	6.0 (4.0, 7.0)	15.0 (12.0, 19.0)	42.0 (29.0, 52.0)
*P*		0.007	< 0.001	< 0.001	< 0.001
Hypertension					
No	328 (19.0)	20.0 (17.0, 23.0)	6.0 (5.0, 8.0)	17.0 (14.0, 22.0)	50.0 (37.0, 63.0)
Yes	1398 (81.0)	19.0 (16.0, 22.0)	6.0 (4.0, 8.0)	16.0 (13.0, 20.0)	45.0 (33.0, 57.0)
*P*		0.003	0.003	< 0.001	< 0.001
Hyperlipidemia					
No	555 (32.2)	19.0 (16.0, 22.0)	6.0 (4.0, 8.0)	16.0 (13.0, 20.0)	46.0 (33.5, 58.0)
Yes	1171 (67.8)	19.0 (16.0, 22.0)	6.0 (4.0, 8.0)	16.0 (13.0, 20.0)	46.0 (34.0, 59.0)
*P*		0.651	0.63	0.884	0.893
Cadmium					
Q1	424 (24.6)	20.0 (16.0, 22.0)	6.0 (4.0, 8.0)	17.0 (13.0, 20.0)	49.0 (36.0, 61.2)
Q2	424 (24.6)	19.0 (16.0, 22.0)	6.0 (4.0, 8.0)	17.0 (13.0, 21.0)	48.0 (35.0, 60.0)
Q3	436 (25.3)	19.0 (16.0, 22.0)	6.0 (4.0, 8.0)	16.0 (13.0, 20.0)	46.0 (34.0, 59.0)
Q4	442 (25.6)	18.0 (16.0, 22.0)	6.0 (4.0, 8.0)	15.0 (12.0, 19.0)	42.0 (32.0, 55.0)
*P*		0.028	0.532	< 0.001	< 0.001
Lead					
Q1	431 (25.0)	19.0 (17.0, 22.0)	6.0 (5.0, 8.0)	16.0 (13.0, 20.0)	48.0 (34.0, 61.0)
Q2	426 (24.7)	20.0 (16.0, 23.0)	6.0 (4.0, 8.0)	17.0 (13.0, 20.0)	49.0 (36.2, 59.0)
Q3	436 (25.3)	19.0 (16.0, 22.0)	6.0 (4.0, 8.0)	16.0 (13.0, 20.0)	47.0 (36.0, 60.0)
Q4	433 (25.1)	18.0 (15.0, 21.0)	6.0 (4.0, 7.0)	16.0 (13.0, 20.0)	42.0 (31.0, 54.0)
*P*		< 0.001	0.003	0.663	< 0.001
Manganese					
Q1	431 (25.0)	19.0 (15.0, 22.0)	6.0 (4.0, 8.0)	17.0 (13.0, 20.0)	44.0 (32.0, 56.0)
Q2	432 (25.0)	19.0 (16.0, 22.0)	6.0 (5.0, 8.0)	16.0 (13.0, 20.0)	46.0 (36.0, 59.0)
Q3	429 (24.9)	19.0 (16.0, 22.0)	6.0 (4.0, 8.0)	16.0 (13.0, 20.0)	47.0 (33.0, 60.0)
Q4	434 (25.1)	19.0 (16.0, 22.0)	6.0 (5.0, 8.0)	16.0 (12.2, 20.0)	48.0 (36.0, 60.0)
*P*		0.402	0.066	0.495	0.01
Mercury					
Q1	421 (24.4)	18.0 (15.0, 21.0)	6.0 (4.0, 7.0)	15.0 (12.0, 19.0)	41.0 (30.0, 54.0)
Q2	441 (25.6)	19.0 (16.0, 22.0)	6.0 (4.0, 7.0)	17.0 (13.0, 20.0)	46.0 (33.0, 58.0)
Q3	432 (25.0)	19.0 (16.0, 22.0)	6.0 (4.0, 8.0)	17.0 (13.0, 20.0)	48.0 (34.0, 60.0)
Q4	432 (25.0)	20.0 (16.0, 23.0)	6.0 (5.0, 8.0)	17.0 (13.0, 21.0)	50.0 (38.0, 63.0)
*P*		< 0.001	< 0.001	0.004	< 0.001
Selenium					
Q1	432 (25.0)	18.0 (15.0, 21.0)	6.0 (4.0, 7.0)	15.0 (12.0, 19.0)	40.0 (28.0, 53.0)
Q2	431 (25.0)	20.0 (16.0, 22.0)	6.0 (4.0, 8.0)	17.0 (13.0, 20.0)	49.0 (34.0, 61.0)
Q3	431 (25.0)	19.0 (16.0, 22.0)	6.0 (5.0, 8.0)	17.0 (13.0, 20.0)	48.0 (37.0, 60.0)
Q4	432 (25.0)	20.0 (16.0, 23.0)	6.0 (4.8, 8.0)	17.0 (13.0, 20.0)	48.0 (35.8, 60.0)
*P*		< 0.001	< 0.001	0.003	< 0.001
DII group					
pro-inflammatory diet	1011 (58.6)	19.0 (15.0, 22.0)	6.0 (4.0, 8.0)	16.0 (12.0, 19.0)	44.0 (31.0, 56.0)
anti-inflammatory diet	715 (41.4)	19.0 (16.5, 23.0)	6.0 (5.0, 8.0)	17.0 (14.0, 21.0)	50.0 (39.0, 63.0)
*P*		0.002	0.008	< 0.001	< 0.001

### Associations between blood trace elements mixture and cognitive performance scores

Table 2 presents the correlation between blood trace element concentration and cognitive function. The study revealed a positive correlation between Se and IRT and DDST scores (β = 2.06, 95%CI:0.7 ~ 3.41; β = 6.41, 95%CI: 2.35 ~ 10.46), respectively. The GLM analysis based on quartiles of the exposure variables demonstrated that Se at Q2 (β = 0.89, 95%CI: 0.33 ~ 1.45)) and Q3 (β = 3.14, 95%CI: 1.46 ~ 4.81)) had the most significant positive impact on cognitive function of IRT and DDST, respectively. Conversely, Pb negatively correlated with IRT scores (β= -0.47, 95%CI: -0.82~-0.11), and the Q3 (β= -0.82, 95%CI: -1.39~-0.25) had the most negative effect. Blood Cd was negatively associated with DSST (β=-1.17, 95%CI: -2.13~-0.22), and the Q3 (β = 3.14, 95%CI: 1.46 ~ 4.81) showed the most significant negative impact.

**Table 2:** Univariate linear regression analysis of the association between trace element exposure and cognitive function scores

Variable	IRT (β(95%CI))	DRT (β(95%CI))	AFT (β(95%CI))	DDST (β(95%CI))
Cadmium				
Cont	-0.31 (-0.64 ~ 0.01)	-0.05 (-0.22 ~ 0.11)	-0.11 (-0.49 ~ 0.27)	-1.17 (-2.13 ~ -0.22)
*P*	0.054	0.533	0.569	0.016
Q1	0(Ref)	0(Ref)	0(Ref)	0(Ref)
Q2	-0.52 (-1.08 ~ 0.05)	-0.06 (-0.35 ~ 0.23)	0.12 (-0.55 ~ 0.78)	-1.91 (-3.59 ~ -0.23)
Q3	-0.23 (-0.8 ~ 0.35)	-0.24 (-0.54 ~ 0.05)	-0.17 (-0.84 ~ 0.51)	-1.87 (-3.57 ~ -0.16)
Q4	-0.72 (-1.34 ~ -0.1)	-0.1 (-0.42 ~ 0.22)	-0.37 (-1.11 ~ 0.36)	-2.33 (-4.18 ~ -0.48)
*P*-t	0.069	0.317	0.244	0.019
Lead				
Cont	-0.47 (-0.82 ~ -0.11)	-0.12 (-0.3 ~ 0.07)	0.19 (-0.24 ~ 0.61)	-0.3 (-1.37 ~ 0.77)
*P*	0.011	0.207	0.389	0.579
Q1	0(Ref)	0(Ref)	0(Ref)	0(Ref)
Q2	-0.2 (-0.76 ~ 0.36)	-0.02 (-0.31 ~ 0.27)	0.06 (-0.6 ~ 0.72)	-0.34 (-2.02 ~ 1.34)
Q3	-0.82 (-1.39 ~ -0.25)	-0.19 (-0.48 ~ 0.1)	0.15 (-0.52 ~ 0.82)	-0.09 (-1.78 ~ 1.6)
Q4	-0.46 (-1.05 ~ 0.14)	-0.14 (-0.45 ~ 0.17)	0.3 (-0.4 ~ 1)	-0.93 (-2.7 ~ 0.84)
*P*-t	0.034	0.222	0.378	0.385
Manganese				
Cont	-0.4 (-0.97 ~ 0.16)	0.07 (-0.22 ~ 0.37)	-0.59 (-1.26 ~ 0.08)	0.39 (-1.3 ~ 2.09)
*P*	0.164	0.622	0.083	0.649
Q1	0(Ref)	0(Ref)	0(Ref)	0(Ref)
Q2	0.06 (-0.49 ~ 0.62)	0.14 (-0.14 ~ 0.43)	-0.65 (-1.31 ~ 0)	0.46 (-1.21 ~ 2.12)
Q3	0.04 (-0.52 ~ 0.6)	0.16 (-0.13 ~ 0.46)	-0.51 (-1.18 ~ 0.15)	-0.34 (-2.02 ~ 1.35)
Q4	-0.1 (-0.68 ~ 0.48)	0.12 (-0.18 ~ 0.42)	-0.81 (-1.49 ~ -0.13)	0.6 (-1.13 ~ 2.32)
*P*-t	0.728	0.422	0.038	0.729
Mercury				
Cont	0.1 (-0.12 ~ 0.31)	0.02 (-0.09 ~ 0.13)	0.12 (-0.14 ~ 0.37)	0.44 (-0.21 ~ 1.08)
*P*	0.377	0.716	0.37	0.183
Q1	0(Ref)	0(Ref)	0(Ref)	0(Ref)
Q2	0.06 (-0.5 ~ 0.62)	0.03 (-0.26 ~ 0.32)	0.43 (-0.23 ~ 1.09)	0.22 (-1.46 ~ 1.9)
Q3	0.28 (-0.3 ~ 0.85)	0.09 (-0.21 ~ 0.39)	0.3 (-0.38 ~ 0.98)	1.34 (-0.38 ~ 3.06)
Q4	0.18 (-0.43 ~ 0.78)	0.18 (-0.13 ~ 0.49)	0.4 (-0.31 ~ 1.11)	1.2 (-0.6 ~ 3)
*P*-t	0.445	0.235	0.353	0.103
Selenium				
Cont	2.06 (0.7 ~ 3.41)	0.58 (-0.12 ~ 1.28)	0.83 (-0.77 ~ 2.43)	6.41 (2.35 ~ 10.46)
*P*	0.003	0.106	0.311	0.002
Q1	0(Ref)	0(Ref)	0(Ref)	0(Ref)
Q2	0.89 (0.33 ~ 1.45)	0.31 (0.02 ~ 0.6)	0.29 (-0.37 ~ 0.95)	2.87 (1.2 ~ 4.53)
Q3	0.49 (-0.07 ~ 1.05)	0.27 (-0.02 ~ 0.56)	0.27 (-0.4 ~ 0.93)	3.14 (1.46 ~ 4.81)
Q4	0.76 (0.19 ~ 1.32)	0.25 (-0.05 ~ 0.54)	0.45 (-0.22 ~ 1.12)	2.54 (0.85 ~ 4.22)
*P*-t	0.042	0.14	0.222	0.004

### Associations between blood trace elements and cognitive performance scores differed by dietary inflammatory index

Subgroup analyses assessed whether an anti-inflammatory or pro-inflammatory diet influenced the relationship between blood trace elements and cognitive function (Figure S2). In the population with a pro-inflammatory diet, blood Cd and IRT showed a negative correlation (β = -0.53, 95%CI:-0.96~-0.11). The Q4 equates (β = -1.15, 95%CI:-1.97~-0.32) demonstrated the most negative effect. The same results were observed between Cd and DSST (β= -1.31, 95%CI:-2.59~-0.03), with all equates showing a negative impact. Conversely, blood Se was positively associated with DSST as a continuous variable (β = 8.57, 95%CI:2.79 ~ 14.36) or quadripartite variable. For IRT, the Q2 equates of blood Se was positively associated. Additionally, the population with an anti-inflammatory diet exhibited negative and positive correlations between blood Pb and Se with IRT, respectively, with β (95% CI) values of -0.72 (-1.28~-0.17) and 2.21 (0.28 ~ 4.15). Q3 is Pb, and Q2 is Se, showing the association.

### Multi-trace element exposures and cognitive function

#### Multi-trace element exposures and cognitive function in the whole population

The BKMR model investigated the association between cognitive function and co-exposure to five blood trace elements. The results showed that the overall effect of the five trace element co-exposure on DRT tended to be positive. However, for IRT, AFT, and DSST, the impact of trace element co-exposure was first reduced and then increased, presenting a negative overall effect (Figure S3). Blood Se emerged as a significant component for improved cognitive performance on IRT and DSST (IRT: 0.401; DSST: 0.617) (Table 3) and presented a positive relationship when all other chemicals were at the 25th, 50th, and 75th percentile levels (Figure S4). Conversely, blood Cd was found to have a negative correlation with DSST and presented vital components for cognitive impairment (PIP: 0.543). The IRT and DRT scores of blood Se showed a linear positive curve, whereas an inverted U-shaped curve was observed in AFT and DSST scores. It was a linear negative curve for blood Cd on IRT and DSST (Figure S5). Additionally, the study found that the slope of the dose-response relationship between Cd and IRT when Se was at the 25th, 50th, and 75th percentile levels indicated the interaction between Cd and DSST. No other interaction was observed between blood Se/Cd and IRT or DSST (Figure S6-9).

**Table 3:** The index from BKMR, WQS and Qg-comp analysis in the whole population, anti-inflammatory diet population and pro-inflammatory diet population

BKMR model	Variable	In the whole population				In anti-inflammatory diet population				In pro-inflammatory diet population			
		IRT	DRT	AFT	DSST	IRT	DRT	AFT	DSST	IRT	DRT	AFT	DSST
	Cadmium	0.052	0.035	0.892	0.543	0.513	0.062	0.399	0.413	0.466	0.089	0.943	0.925
	Lead	0.117	0.098	0.696	0.819	0.777	0.141	0.250	0.384	0.019	0.416	0.520	0.421
	Manganese	0.013	0.014	0.177	0.127	0.175	0.079	0.159	0.209	0.019	0.016	0.472	0.096
	Mercury	0.051	0.023	0.888	0.097	0.800	0.247	0.285	0.986	0.002	0.023	0.464	0.013
	Selenium	0.401	0.010	0.615	0.617	0.509	0.112	0.209	0.327	0.525	0.058	0.507	0.795
**Qg-comp model**	Variable	In the whole population				In anti-inflammatory diet population				In pro-inflammatory diet population			
		IRT	DRT	AFT	DSST	IRT	DRT	AFT	DSST	IRT	DRT	AFT	DSST
	Cadmium	-0.402	-0.438	-0.404	-0.837	0.064	0.323	-0.145	0.606	-0.767	-0.885	-0.671	-1.000
	Lead	-0.546	-0.562	0.343	-0.163	-0.851	-1.000	0.490	0.394	-0.100	-0.115	0.040	0.041
	Manganese	-0.053	0.248	-0.596	0.107	-0.149	0.205	-0.353	0.023	-0.133	0.110	-0.302	0.081
	Mercury	0.293	0.368	0.314	0.327	0.475	0.055	-0.502	0.371	0.148	0.486	0.960	0.295
	Selenium	0.707	0.385	0.342	0.565	0.462	0.417	0.510	0.606	0.852	0.404	-0.028	0.583
**WQS** **model**	Variable	In the whole population				In anti-inflammatory diet population				In pro-inflammatory diet population			
		IRT(+)	DRT(+)	AFT(+)	DSST(+)	IRT(+)	DRT(+)	AFT(+)	DSST(+)	IRT(+)	DRT(+)	AFT(+)	DSST(+)
	Cadmium	0.035	0.039	0.019	0.004	0.235	0.289	0.074	0.133	0.001	0.018	0.000	0.002
	Lead	0.012	0.122	0.531	0.171	0.018	0.006	0.360	0.015	0.127	0.232	0.280	0.200
	Manganese	0.156	0.453	0.032	0.184	0.039	0.096	0.030	0.032	0.009	0.020	0.326	0.023
	Mercury	0.053	0.087	0.139	0.058	0.584	0.453	0.054	0.516	0.095	0.454	0.294	0.379
	Selenium	0.744	0.299	0.279	0.583	0.124	0.156	0.482	0.304	0.769	0.275	0.101	0.396
	Index	0.379 (0.073 ~ 0.686)	0.207 (-0.004 ~ 0.417)	0.151 (-0.296 ~ 0.597)	0.805 (-0.269 ~ 1.879)	0.094 (-0.471 ~ 0.660)	-0.068 (-0.384 ~ 0.249)	0.764 (0.055 ~ 1.473)	-0.311 (-2.012 ~ 1.390)	0.320 (-0.062 ~ 0.701)	0.190 (-0.086 ~ 0.467)	0.048 (-0.642 ~ 0.739)	1.752 (0.231 ~ 3.274)
	Variable	In the whole population				In anti-inflammatory diet population				In pro-inflammatory diet population			
		IRT(-)	DRT(-)	AFT(-)	DSST(-)	IRT(-)	DRT(-)	AFT(-)	DSST(-)	IRT(-)	DRT(-)	AFT(-)	DSST(-)
	Cadmium	0.340	0.347	0.434	0.716	0.075	0.035	0.254	0.085	0.500	0.441	0.701	0.590
	Lead	0.236	0.189	0.016	0.052	0.428	0.685	0.055	0.550	0.104	0.113	0.052	0.070
	Manganese	0.142	0.049	0.321	0.039	0.315	0.154	0.333	0.324	0.240	0.302	0.047	0.287
	Mercury	0.246	0.308	0.152	0.190	0.021	0.014	0.326	0.003	0.130	0.054	0.040	0.023
	Selenium	0.037	0.108	0.077	0.003	0.162	0.112	0.031	0.037	0.026	0.090	0.160	0.030
	Index	-0.029 (-0.482 ~ 0.424)	0.137 (-0.095 ~ 0.368)	-0.316 (-0.797 ~ 0.165)	-0.613 (-1.609 ~ 0.382)	0.319 (-0.934 ~ 0.297)	-0.053 (-0.305 ~ 0.198)	0.223 (-0.57 ~ 1.015)	-0.070 (-1.678 ~ 1.538)	-0.043 (-0.604 ~ 0.518)	0.058–0.239 ~ 0.355)	-0.046 (-1.358 ~ 0.475)	-0.676 (-2.163 ~ 0.810)

Furthermore, the WQS index was calculated to evaluate the overall effect of trace element mixture and cognitive function (Table 3). The fully adjusted WQS model revealed a significant positive association between the WQS index and IRT (0.379 (0.073 ~ 0.686)). Se had the highest weight (0.744) amongst all chemicals in the WQS index. Results from the favorable WQS model analysis further supported the beneficial effect of Se on IRT. In contrast, the research revealed no statistically significant discrepancies in the negative WQS model and other cognitive function scores. The weight study confirmed that Cd was most negatively correlated with IRT and DSST, while Se was most positively correlated with DSST (Figure S10). Qg-comp model (Figure S11) also supported these results. Se had the highest positive correlation with IRT and DSST, with weights of 0.707 and 0.565, respectively. Cd and Pb showed the highest negative correlation with IRT with weights of 0.402 and 0.546, respectively, whereas Cd weighted 0.837 on DSST.

#### Multi-trace element exposures and cognitive function in an anti-inflammatory diet and pro-inflammatory diet population

**Fig. 2:**
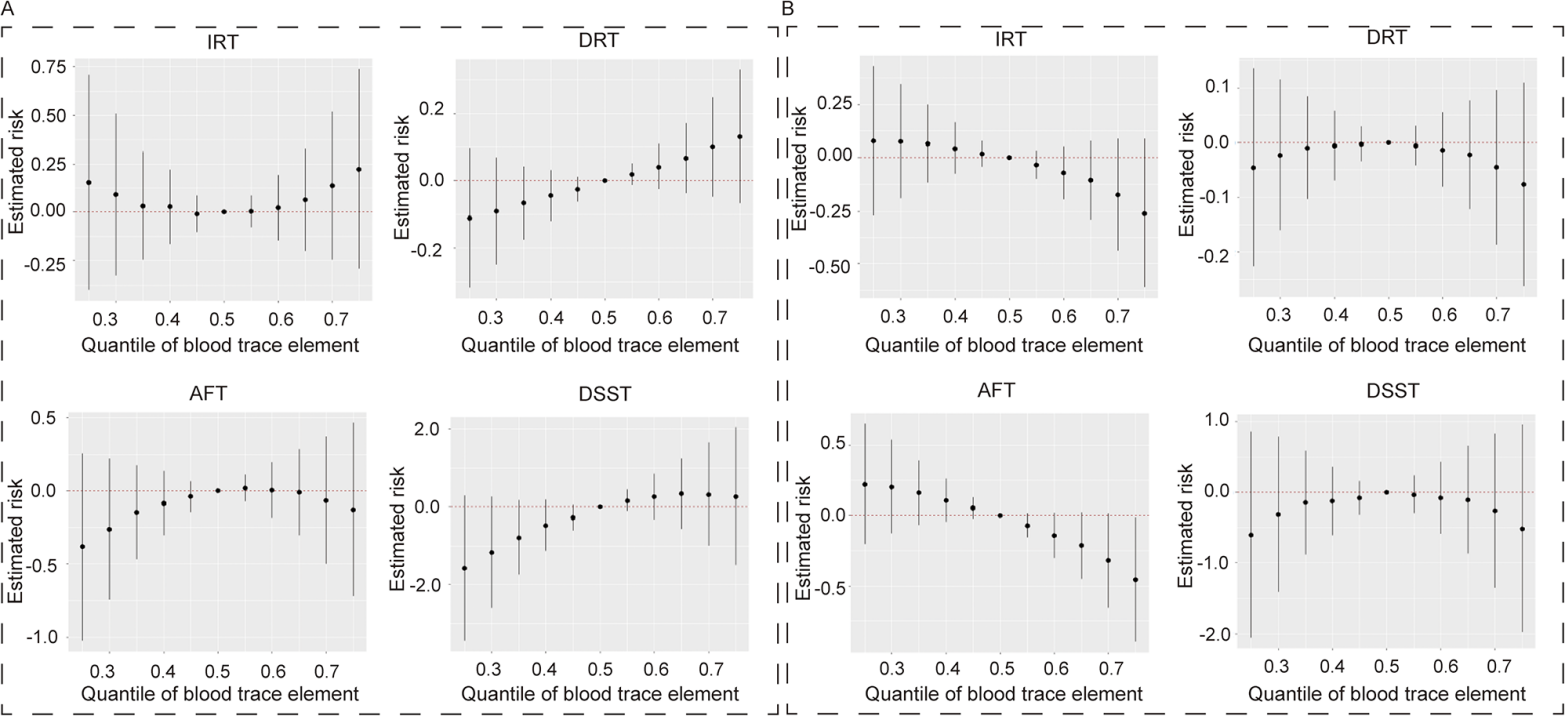
Joint effect (95% CI) of the trace elements on IRT, DRT, AFT, and DSST when all the trace elements at particular percentiles were compared to those at their 50th percentile in the anti-inflammatory (**A**) and pro-inflammatory diet (**B**) by the BKMR model. The model was adjusted for adjusted by sex, age, race, education, income, marital status, physical activity, drinking, smoking, BMI, diabetes, hypertension, and hyperlipidemia

**Fig. 3:**
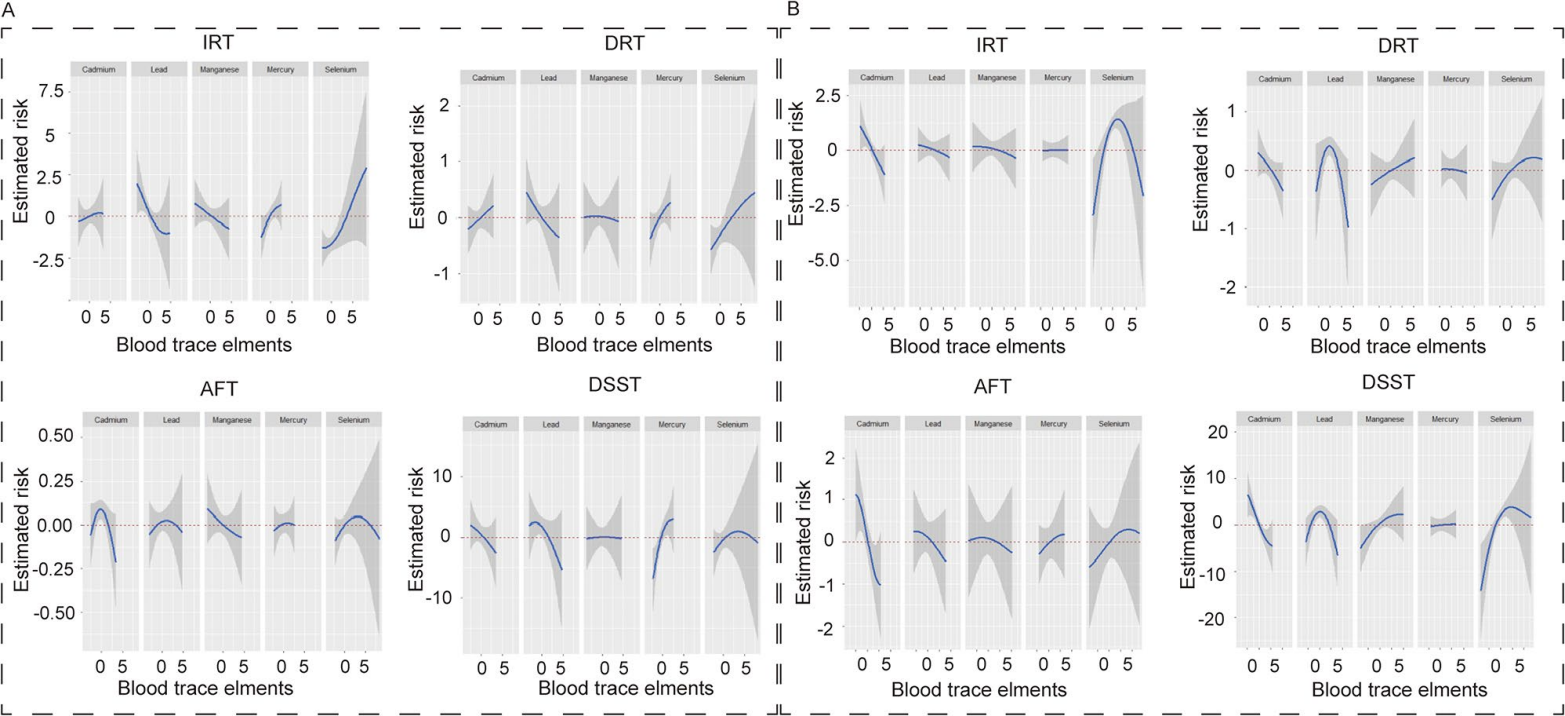
Univariate exposure-response relationships (95% CI) between the trace element and cognitive performance tests (IRT, DRT, AFT, and DSST) while fixing at the median for other four trace element concentrations in the anti-inflammatory (**A**) and pro-inflammatory diet (**B**) by the BKMR model. The model was adjusted for sex, age, race, education, income, marital status, physical activity, drinking, smoking, BMI, diabetes, hypertension, and hyperlipidemia

The influence of diet on trace elements and cognitive function was studied using the BKMR model. Specifically, the study examined the correlation between blood Cd and Se with IRT and DSST. The results revealed that the effects of trace element co-exposure on IRT and DSST were significantly different under anti-inflammatory and pro-inflammatory diets. Under an anti-inflammatory diet, trace element co-exposure’s overall impact on IRT was opposite to a positive effect, while DSST showed a linear positive correlation (Fig. 2A). In contrast, under a pro-inflammatory diet, the overall impact of trace element co-exposure on IRT was negatively correlated, while for DSST, it was the same as that of the whole population, which was reduced firstly and then increased and presented a negative effect overall (Fig. 2B). Blood Cd emerged as the significant negative contributor to IRT and DSST in the pro-inflammatory diet, with PIP values of 0.466 and 0.925 (Table 3). Blood Se was a substantial component for enhancing cognitive performance on IRT and DSST in the pro-inflammatory diet, not in the anti-inflammatory diet (Figure S12-S13). The exposure-response trends for each trace element are shown in Fig. 3. The IRT scores of blood Se showed a linear positive curve in the anti-inflammatory diet, whereas an inverted U-shaped curve in the pro-inflammatory diet. The inverted U-shaped curve was also observed in the DSST scores of blood Se in anti-inflammatory and pro-inflammatory diets. Blood Cd on DSST exhibited a linear positive curve in anti-inflammatory or pro-inflammatory diets. Furthermore, the interactions between Cd and Mn, Pb and Hg were observed in the pro-inflammatory diet (Figure S14-S21).

**Fig. 4:**
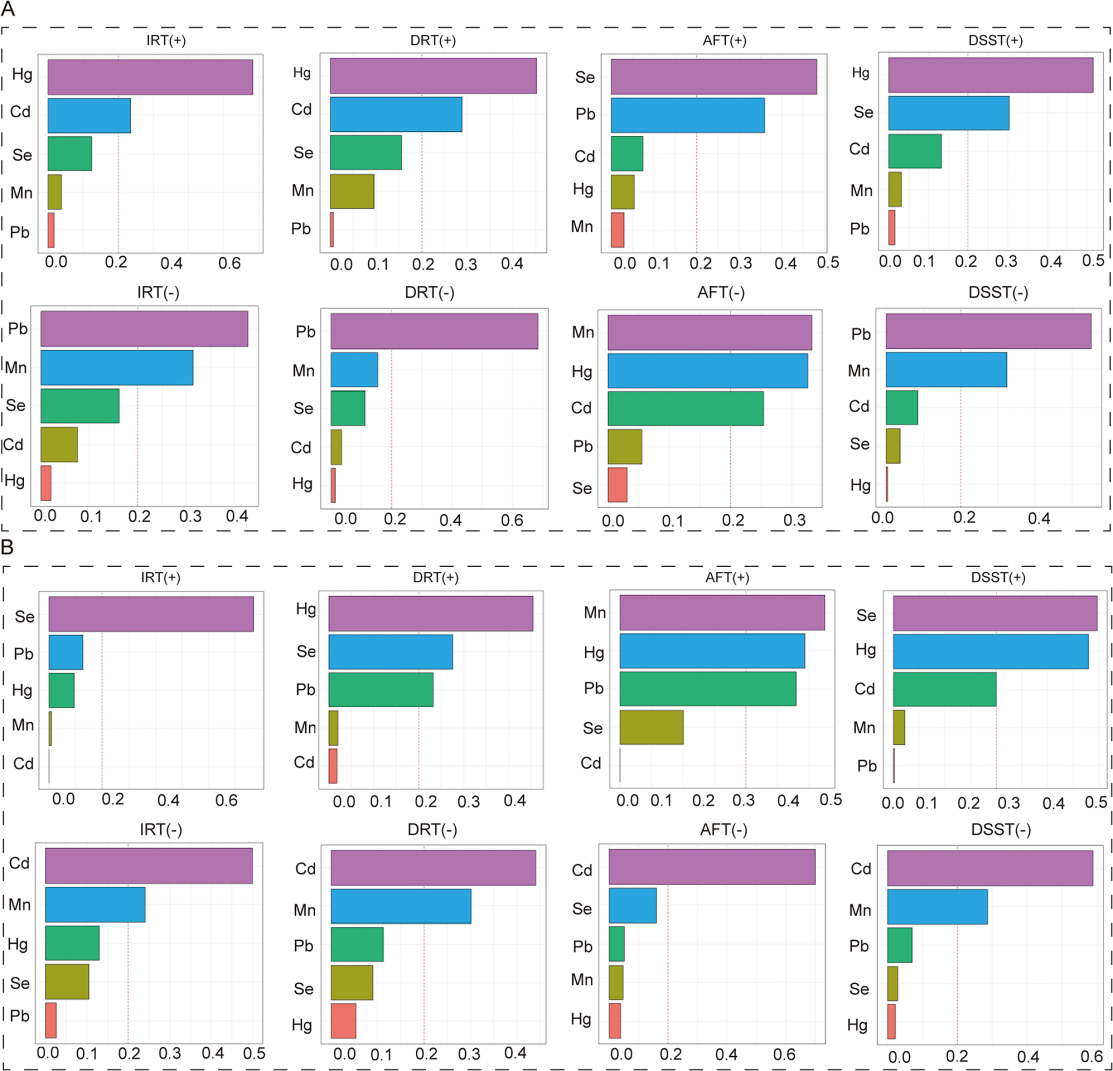
WQS model regression index weights for blood trace elements and cognitive function in the anti-inflammatory (**A**) and pro-inflammatory diet (**B**). The model was adjusted for sex, age, race, education, income, marital status, physical activity, drinking, smoking, BMI, diabetes, hypertension, and hyperlipidemia

**Fig. 5:**
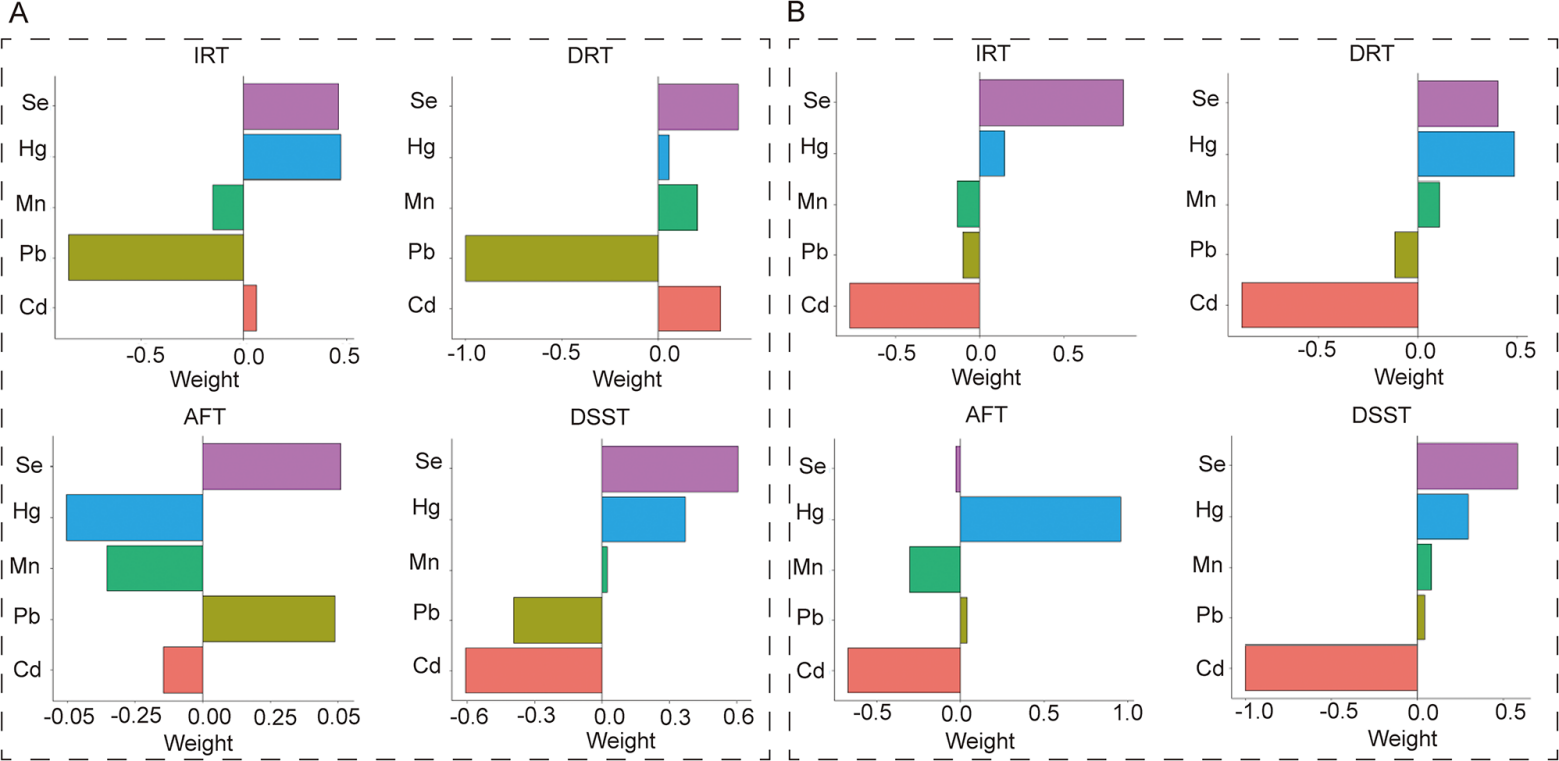
Qg-comp model regression index weights for blood trace elements and cognitive function in the anti-inflammatory (**A**) and pro-inflammatory diet (**B**). The model was adjusted for sex, age, race, education, income, marital status, physical activity, drinking, smoking, BMI, diabetes, hypertension, and hyperlipidemia

The WQS and Qg-comp models were used to determine the correlation between co-exposure trace elements and cognitive function. Positive and negative contributors were identified. The WQS index was significantly associated with DSST (1.752(0.231–3.274)) in the pro-inflammatory diet. The chemical with the highest weight was Se, with a weight of 0.396. The pro-inflammatory diet further supported the beneficial effects of blood Se on DSST. However, no meaningful variations were found in the other WQS index. The negative contributor for IRT and DSST was Cd (WQS index: 0.500 for IRT, 0.590 for DSST), while the positive contributor was Se (WQS index: 0.769 for IRT, 0.396 for DSST). Cd showed the most negative correlation with IRT and DSST, while Se showed the most positive correlation with IRT and DSST in the pro-inflammatory diet (Fig. 4). The Qg-comp model confirmed the positive correlation between Se and IRT or DSST and the negative association between Cd and IRT or DSST in the pro-inflammatory diet (Fig. 5).

### Sensitivity analysis

The sensitivity analyses conducted in this study have demonstrated that blood Se remains significantly associated with IRT and DSST, while blood Cd exhibits a negative correlation with DSST across three models that adjust for different confounding variables. These findings suggested the robustness and reliability of our results. Furthermore, the consistency of the results was maintained even after adjusting for the confounding effect of the other four trace elements, as evidenced by the data presented in Table S2.

## Discussion

Some trace elements, especially heavy metals, have been identified as potent neurotoxins that could cause acute and chronic neurotoxicity, resulting in cognitive function impairment. On the other hand, diet is a crucial factor in the development of many neurological disorders. Our study aimed to determine if dietary modifications could affect the association between blood trace element concentrations and cognitive performance test scores in older adults. Our study represents a pioneering epidemiological investigation characterized by its large-scale nature and the utilization of diverse statistical models to explore the effect of trace elements on cognitive function, both individually and in combination with one another. The study focuses on modifying dietary intake to elucidate the extent of the influence of trace element exposure on cognitive function. To the best of our knowledge, no other study has employed such an approach, making our research significantly contribute to the existing literature on the subject.

The findings of a negative association between Cd exposure and cognitive performance are consistent with previous studies [25, 26]. Cd exhibits neurotoxic effects by inducing oxidative stress and neuroinflammation [27]. Exposure to Cd, whether at high levels for a short duration or low levels for a prolonged period, stimulates the production of reactive oxygen species by disrupting mitochondrial function and depleting antioxidants. The phenomenon leads to the onset of oxidative stress in neuronal and brain endothelial cells, resulting in impaired neurodevelopment and oxidative stress-dependent neuroinflammation [28]. We also found a negative association between Pb exposure and cognitive function, aligning with the established literature on Pb neurotoxicity. Pb can activate apoptotic pathways, including mitochondrial dysfunction, cytosolic calcium overload, and activation of caspases [9]. Pb induces oxidative stress, neuroinflammation, and alterations in neurotransmitter systems, which can disrupt cognitive processes such as memory, attention, and processing speed [29, 30].

In contrast, our results suggest a protective effect of Se on cognitive function, particularly in the context of a pro-inflammatory diet. This finding aligns with previous research indicating that Se, an essential trace element with antioxidant properties, may help to mitigate oxidative stress and inflammation in the brain [31, 32]. However, physiological aging may decrease the absorption and utilization of selenium [33], and thus, the potential risk of selenium deficiency cannot be overlooked in this population. Overall, while the observed concentrations were comparable to the daily recommended intake, the unique physiological condition of the elderly warrants consideration of potential nutritional concerns when interpreting the results [34]. Additionally, it is essential to acknowledge the ongoing scientific debate regarding Se’s role as an antioxidant. While our study focused on the potential beneficial effects of Se in mitigating the impact of trace element exposure on cognitive function, recent animal and human studies have questioned the antioxidant properties of Se [35, 36]. These findings suggest that our interpretation of Se’s protective effects may need re-evaluation in light of emerging evidence. Furthermore, excessive Se exposure or overexposure can have adverse effects, including an increased risk of developing neurological disorders such as dementia [37–39].

An important finding from our study was the presence of non-linear associations between certain trace elements and cognitive function scores. The inverted U-shaped relationship between Se levels and cognitive scores (AFT, DSST) suggests that Se deficiency and excess may adversely impact cognitive function due to Se’s role in redox homeostasis and neuroprotective mechanisms [40]. Similarly, the inverted U-shaped association between Pb and DSST scores supported the notion that low and high Pb exposures may harm cognitive performance [41]. These non-linear associations highlight the complexity of the relationships between trace element exposures and cognitive function and underscore the importance of considering potential non-monotonic effects. Further research is needed to elucidate the mechanisms underlying these non-linear associations and to determine optimal exposure levels for essential trace elements like Se.

The modifying effect of dietary inflammatory potential on the associations between trace elements and cognitive function is a novel finding of our study. Previous research has shown that diets high in pro-inflammatory nutrients, such as saturated and trans fats, and low in anti-inflammatory nutrients, such as fruits, vegetables, and whole grains, are associated with increased risk of cognitive decline and dementia [42, 43]. Our results suggest that a pro-inflammatory diet may exacerbate the adverse effects of Cd exposure on cognitive health, while an anti-inflammatory diet may attenuate these effects. The biological mechanisms underlying these interactions are not fully understood but may involve the interplay between oxidative stress, inflammation, and neurotoxicity.

However, it is essential to note that while the DII reflects overall dietary inflammatory potential, it is derived from studies examining individual nutrient effects rather than complete dietary patterns. Evaluating associations between specific nutrients and cognitive outcomes may underestimate the genuine relationship compared to analyzing whole dietary patterns in conjunction with trace element exposures. Future studies should consider the complex interactions between dietary patterns, trace element exposures, and cognitive health to understand the underlying mechanisms better and develop targeted interventions.

In addition, our study found no significant associations between Hg and Mn exposures and cognitive function tests. While some previous studies have reported neurotoxic effects of Hg and Mn exposure [44, 45], others have found inconsistent or null results [46]. The discrepancies in findings may be due to differences in exposure levels, study populations, and cognitive assessment methods. Further research is needed to clarify the potential impact of these trace elements on cognitive function in elderly adults.

The present study utilized multiple statistical models (GLM, BKMR, WQS, and Qg-comp) to investigate the individual and joint effects of trace element exposures on cognitive function and the modifying role of dietary inflammatory potential. Using multiple models enhanced the reliability of the results compared with that of a single model [47]. Furthermore, the models were meticulously adjusted for covariates that could influence trace elements and cognitive function, minimizing the risk of confounding bias. A comprehensive assessment of cognitive function was undertaken by employing four tests (IRT, DRT, AFT, and DSST) to analyze the relationship between trace element exposure and cognitive function under different diet statuses. In the future, utilizing natural products and nanotechnological approaches to counter trace element toxicity may provide new therapeutic avenues to mitigate the detrimental effects of trace element exposure on cognitive function [9].

However, this study has certain limitations. Firstly, it is a cross-sectional study, thus lacking longitudinal follow-up of cognitive status and trace element concentrations, and hence, cannot establish a causal relationship between mixed trace elements and cognitive function under DII. Secondly, Fixed values used to replace levels of trace elements below the detection limit may have underestimated the association effect. Therefore, while the study provides valuable insights, it’s necessary to conduct more prospective cohort studies in the future to confirm the association.

## Conclusion

This study provides evidence for the complex interplay between trace element exposures, dietary inflammatory potential, and cognitive function in elderly adults in the United States. We found that higher Cd levels were associated with poorer memory and processing speed, particularly with a pro-inflammatory diet, while higher Se levels were associated with better cognitive performance, especially in a pro-inflammatory diet. These findings suggest that dietary quality may modify the impact of trace element exposures on cognitive health in older adults. Future research should also consider a broader range of environmental exposures and dietary factors and their potential interactions with lifestyle factors. Public health strategies should aim to reduce exposure to neurotoxic trace elements and promote anti-inflammatory diets to mitigate cognitive decline in older adults.

## Electronic supplementary material

Below is the link to the electronic supplementary material.

Supplementary Material 1

## Data Availability

The datasets generated and/or analysed during the current study are available in the NHANES (https://www.cdc.gov/nchs/nhanes/).

## Abbreviations

GLM: Generalized Linear Regression Model
BKMR: Bayesian Kernel Machine Regression Model
WQS: Weighted Quantile Sum Regression
Qg-comp: Quantile g-computation regression analysis
Mn: Manganese
Pb: Lead
Cd: Cadmium
Hg: Total Mercury
Se: Selenium
DII: Dietary Inflammatory Index
NHANES: National Health and Nutrition Examination Survey
CDC: Centers for Disease Control and Prevention
NCHS: National Center for Health Statistics
IRT: Instant Recall Test
DRT: Delayed Recall Test
AFT: Animal Fluency Test
DSST: Digit Symbol Substitution Test
ICP-MS: Inductively coupled plasma mass spectrometry
LLOD: Lower Limit Of Detection
MET: Metabolic Equivalent of Task
IQR: Interquartile Ranges
