# Supplementary material for: Uncovering the relationship between trace element exposure, cognitive function, and dietary inflammation index in elderly americans from the National Health and Nutrition Examination Survey 2011–2014
Source: BMC Public Health. 2024 Sep 16;24:2516. doi: 10.1186/s12889-024-20060-4 (PMC11403966; doi:10.1186/s12889-024-20060-4)
Supplement: Supplementary file 1 — Supplementary Material 1 [file 12889_2024_20060_MOESM1_ESM.docx]

**Uncovering the Relationship between Trace Element Exposure, Cognitive Function, and Dietary Inflammation Index in Elderly Americans from the National Health and Nutrition Examination Survey 2011-2014**

**Chunlan Tang^1,2^, Min Shen^3^, Hang Hong^2,*^**

^1^ Department of Ophthalmology, The Affiliated People’s Hospital of Ningbo

University, Ningbo, Zhejiang, 315040, China

^2^ School of Public Health, Health Science Center, Ningbo University, Ningbo, Zhejiang, 315211, China

^3^ Reference Laboratory, Medical System Biotechnology Co., Ltd. Ningbo, Zhejiang, 315104, China

^*^ Corresponding author:

School of Public Health, Health Science Center, Ningbo University, Ningbo, Zhejiang, 315211, China

E-mail address: honghang28@163.com (H.Hong)

Table S1 Descriptive statistical results of the five trace elements in the population were included

| Trace elements | Mean | Percentile | | | Normtest.W^a^ | Normtest.p |
| --- | --- | --- | --- | --- | --- | --- |
|  |  | 25th | 50th | 75th |  |  |
| Cadmium | 0.51 | 0.25 | 0.37 | 0.59 | 0.72 | <0.001 |
| Lead | 1.87 | 1.02 | 1.47 | 2.21 | 0.65 | <0.001 |
| Manganese | 9.40 | 6.96 | 8.74 | 11.02 | 0.74 | <0.001 |
| Mercury | 1.65 | 0.48 | 0.91 | 1.88 | 0.60 | <0.001 |
| Selenium | 195.71 | 177.92 | 193.32 | 208.46 | 0.70 | <0.001 |

^a^ The statistic of a Shapiro–Wilk test of normality (normtest.W) and its associated probability (normtest.p)

Table S2 Univariate linear regression analysis of the association between Se, Cd and DSST scores, Se and IRT scores.

| Variable | DSST | | | | |
| --- | --- | --- | --- | --- | --- |
| Selenium | Model 1 | Model 2 | Model 3 | Model 4 | Model 5 |
| Continuous | 15.26 (9.85~20.67) | 14.46 (9.23~19.7) | 7.18 (3.08~11.29) | 6.18 (2.11~10.26) | 6.41 (2.35~10.46) |
| P | <0.001 | <0.001 | 0.001 | 0.003 | 0.002 |
| Q1 | 0(Ref) | 0(Ref) | 0(Ref) | 0(Ref) | 0(Ref) |
| Q2 | 6.33 (4.07~8.58) | 5.8 (3.63~7.97) | 3.1 (1.41~4.8) | 2.95 (1.28~4.62) | 2.87 (1.2~4.53) |
| Q3 | 7.21 (4.95~9.46) | 6.65 (4.48~8.83) | 3.62 (1.92~5.32) | 3.29 (1.61~4.97) | 3.14 (1.46~4.81) |
| Q4 | 6.5 (4.25~8.75) | 6.3 (4.12~8.47) | 2.83 (1.11~4.54) | 2.43 (0.73~4.12) | 2.54 (0.85~4.22) |
| P-t | <0.001 | <0.001 | 0.001 | 0.006 | 0.004 |
| Cadmium | DSST | | | | |
| Continuous | -2.51 (-3.66~-1.35) | -2.54 (-3.65~-1.43) | -1.21 (-2.11~-0.32) | -1.01 (-1.97~-0.05) | -1.17 (-2.13~-0.22) |
| P | <0.001 | <0.001 | 0.008 | 0.04 | 0.016 |
| Q1 | 0(Ref) | 0(Ref) | 0(Ref) | 0(Ref) | 0(Ref) |
| Q2 | -1.12 (-3.41~1.18) | -1.02 (-3.23~1.19) | -1.69 (-3.4~0.01) | -1.71 (-3.4~-0.02) | -1.91 (-3.59~-0.23) |
| Q3 | -2.04 (-4.31~0.24) | -1.78 (-3.98~0.43) | -1.65 (-3.36~0.07) | -1.58 (-3.3~0.13) | -1.87 (-3.57~-0.16) |
| Q4 | -4.68 (-6.95~-2.41) | -4.52 (-6.71~-2.32) | -2.36 (-4.12~-0.61) | -1.98 (-3.84~-0.12) | -2.33 (-4.18~-0.48) |
| P-t | <0.001 | <0.001 | 0.013 | 0.049 | 0.019 |
| Selenium | IRT | | | | |
| Continuous | 3.13 (1.68~4.58) | 2.11 (0.76~3.45) | 2.01 (0.66~3.37) | 2.06 (0.7~3.41) | 1.95 (0.58~3.31) |
| *P* | <0.001 | 0.002 | 0.004 | 0.003 | 0.005 |
| Q1 | 0(Ref) | 0(Ref) | 0(Ref) | 0(Ref) | 0(Ref) |
| Q2 | 1.34 (0.74~1.95) | 0.89 (0.33~1.45) | 0.89 (0.34~1.45) | 0.89 (0.33~1.45) | 0.89 (0.32~1.45) |
| Q3 | 1.03 (0.43~1.64) | 0.53 (-0.03~1.09) | 0.5 (-0.06~1.06) | 0.49 (-0.07~1.05) | 0.47 (-0.1~1.03) |
| Q4 | 1.22 (0.61~1.82) | 0.78 (0.22~1.34) | 0.74 (0.17~1.31) | 0.76 (0.19~1.32) | 0.73 (0.16~1.3) |
| *P*-t | 0.001 | 0.031 | 0.047 | 0.042 | 0.058 |

Model 1:Crude model

Model 2:adjusted by sex, age, race, education, income, marital status.

Model 3:adjusted by sex, age, race, education, income, marital status, physical activity, drinking, smoking, BMI, DII.

Model 4:adjusted by sex, age, race, education, income, marital status, physical activity, drinking, smoking, BMI, DII, diabetes, hypertension, hyperlipidemia.

Model 5:adjusted by sex, age, race, education, income, marital status, physical activity, drinking, smoking, BMI, DII, diabetes, hypertension, hyperlipidemia, and other four trace elements


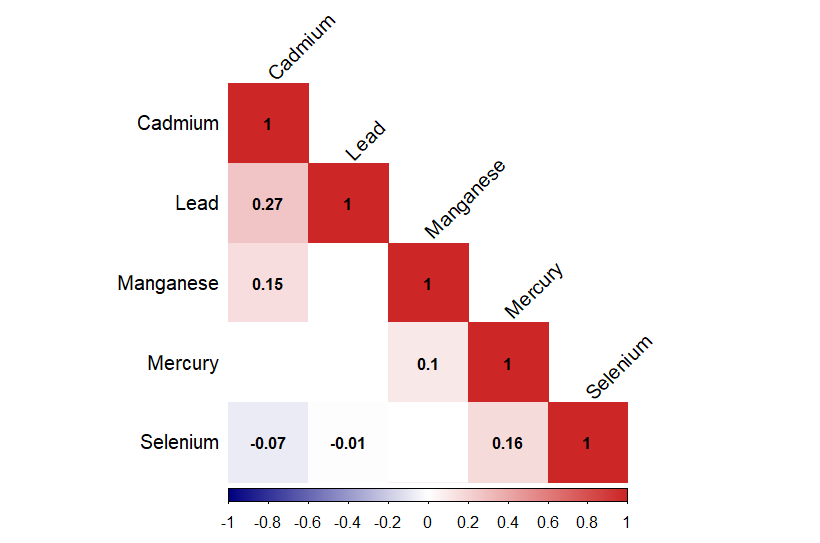


Fig S1 Pearson correlations between ln-transformed concentrations of five trace elements


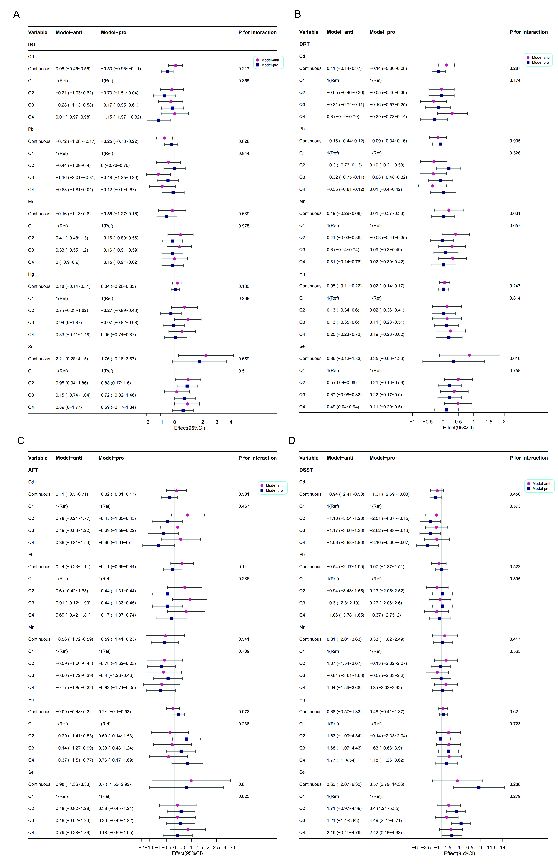


Fig S2 Associations between blood trace elements (continuous and quartile variables) and four cognitive performance tests in the anti-inflammatory and pro-inflammatory diet. The model was adjusted for adjusted by sex, age, race, education, income, marital status, physical activity, drinking, smoking, BMI, DII, diabetes, hypertension, and hyperlipidemia.


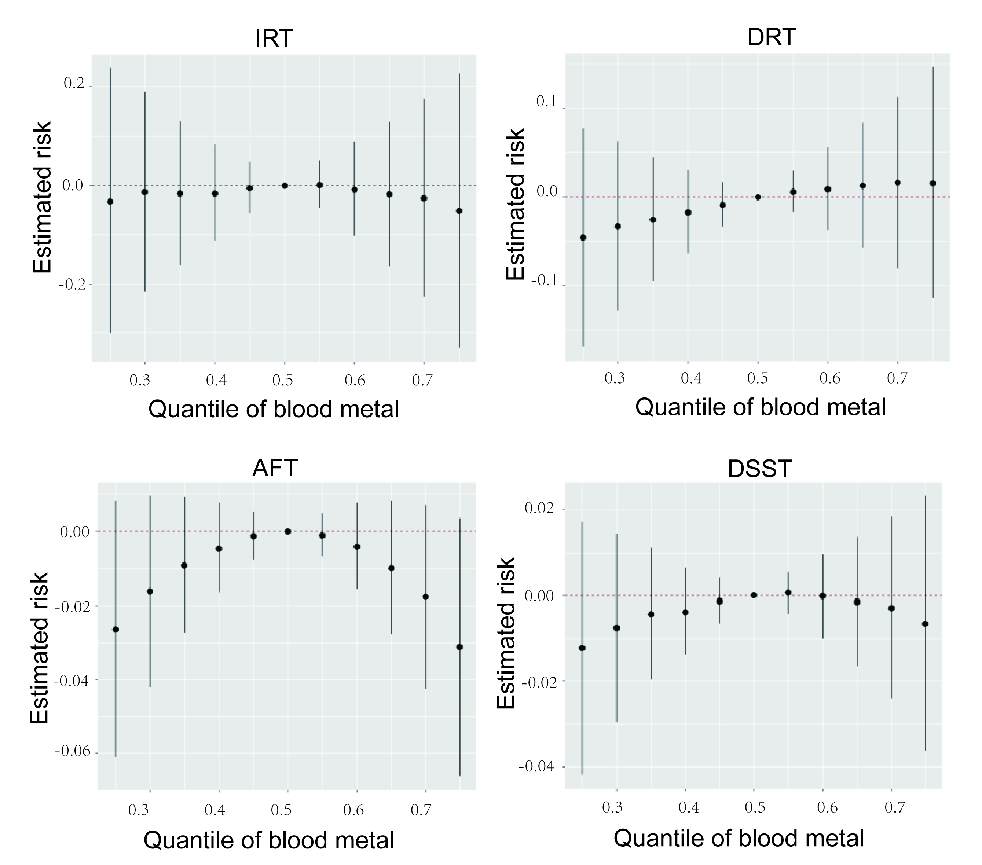


Fig S3 Joint effect (95% CI) of the trace elements on IRT, DRT, AFT, and DSST when all the trace elements at particular percentiles were compared to those at their 50th percentile in the whole population by the BKMR model. The model was adjusted for adjusted by sex, age, race, education, income, marital status, physical activity, drinking, smoking, BMI, diabetes, hypertension, hyperlipidemia, and DII.


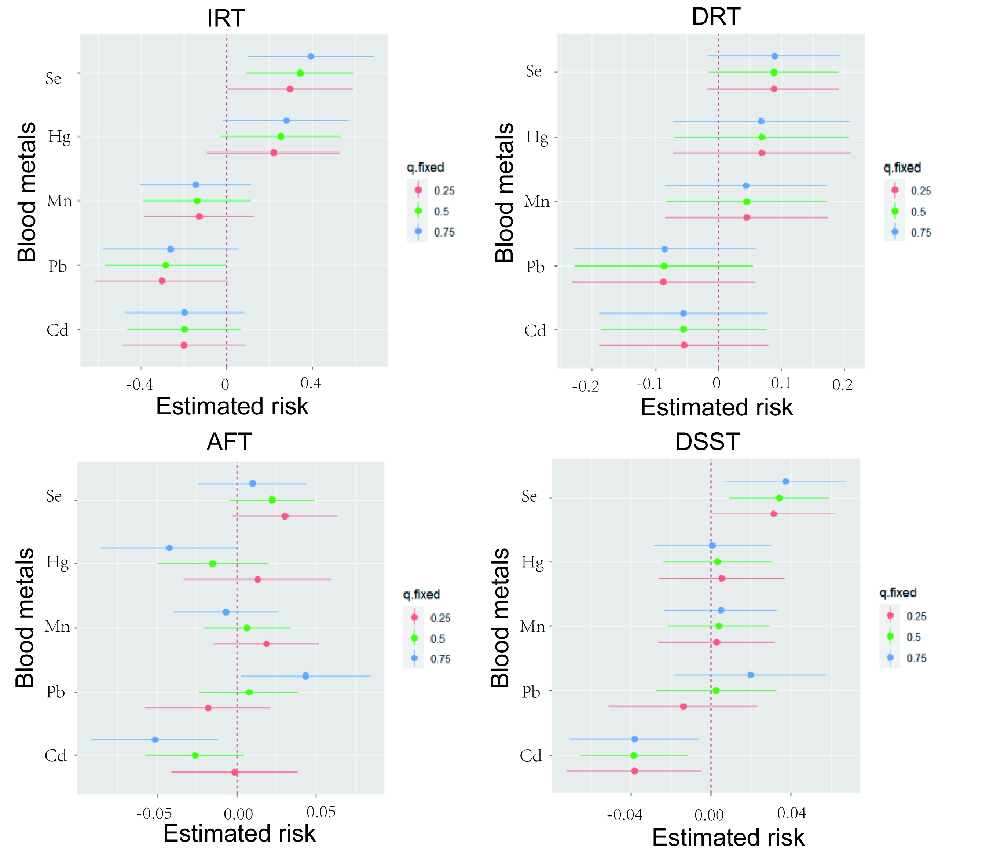


Fig S4 The effect of a single trace element variable at the 25th to 75th percentile on the IRT, DRT, AFT, and DSST by BKMR model analysis in the whole population when other trace elements were fixed at the 25th, 50th, and 75th percentiles.


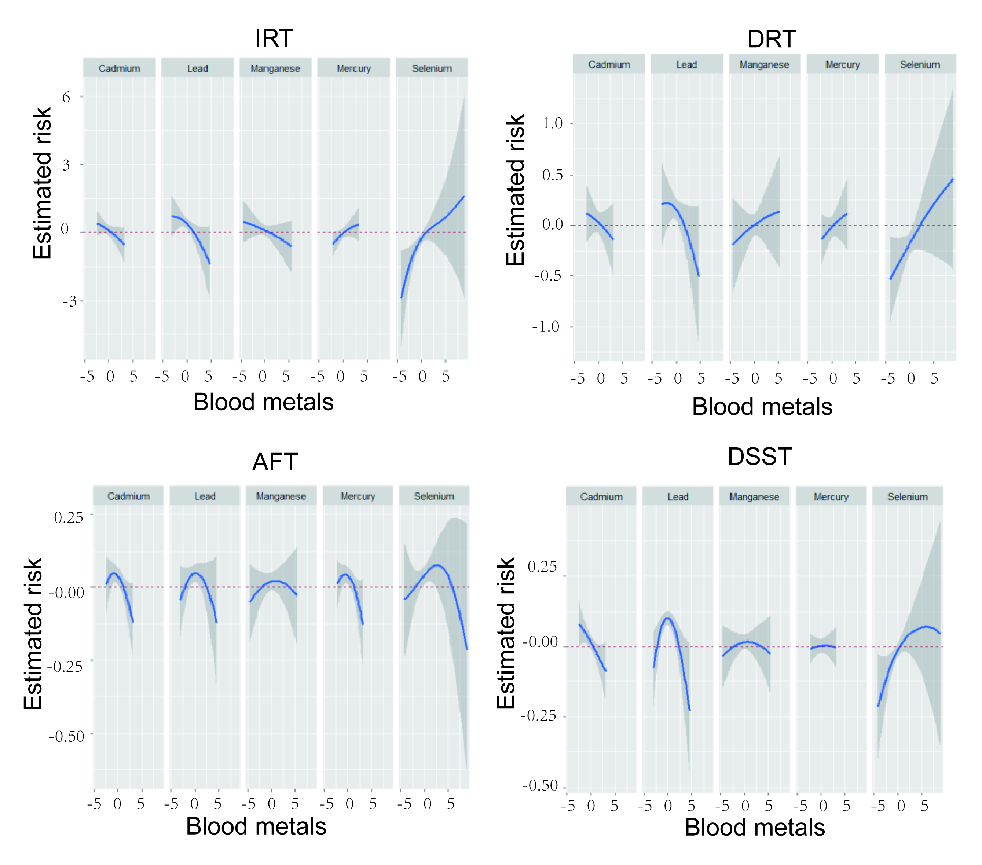


Fig S5 Univariate exposure-response relationships (95% CI) between the trace element and cognitive performance tests (IRT, DRT, AFT, and DSST) while fixing at the median for other four trace element concentrations in the whole population by the BKMR model. The model was adjusted for adjusted by sex, age, race, education, income, marital status, physical activity, drinking, smoking, BMI, diabetes, hypertension, hyperlipidemia, and DII.


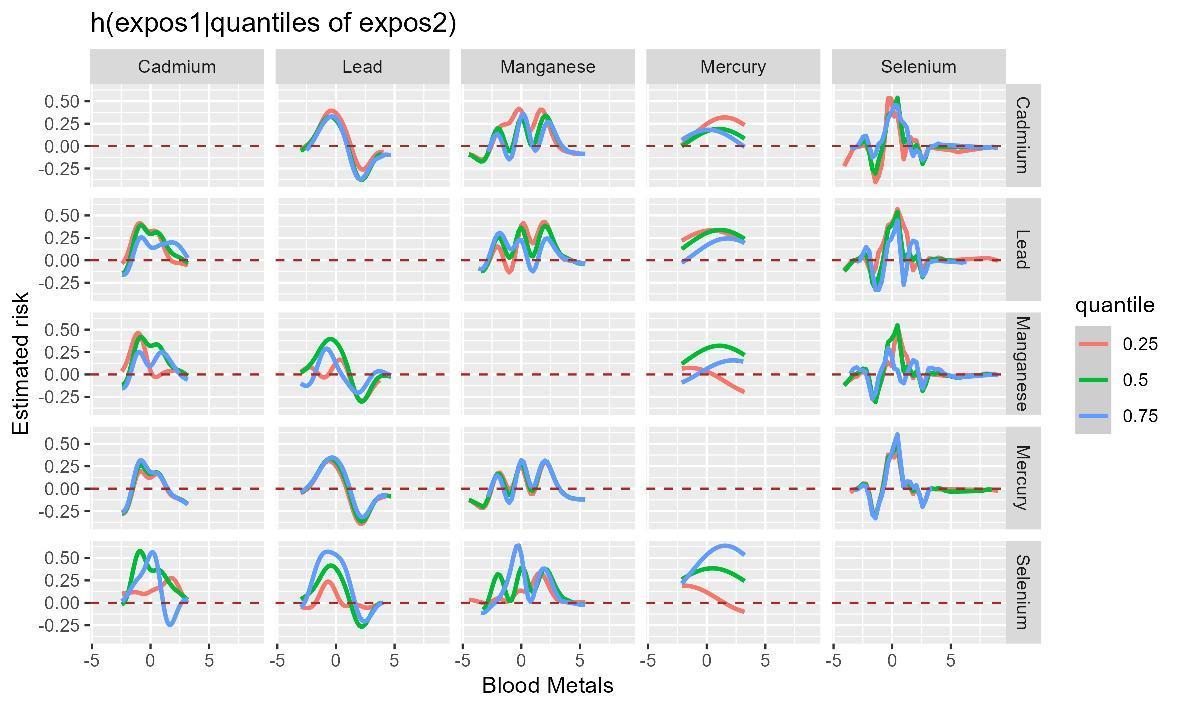


Fig S6 Bivariate exposure-response relationship for each trace element on IRT when the other trace elements were fixed at 25th, 50th, and 75th in the whole population by the BKMR model. The model was adjusted for adjusted by sex, age, race, education, income, marital status, physical activity, drinking, smoking, BMI, diabetes, hypertension, hyperlipidemia, and DII.


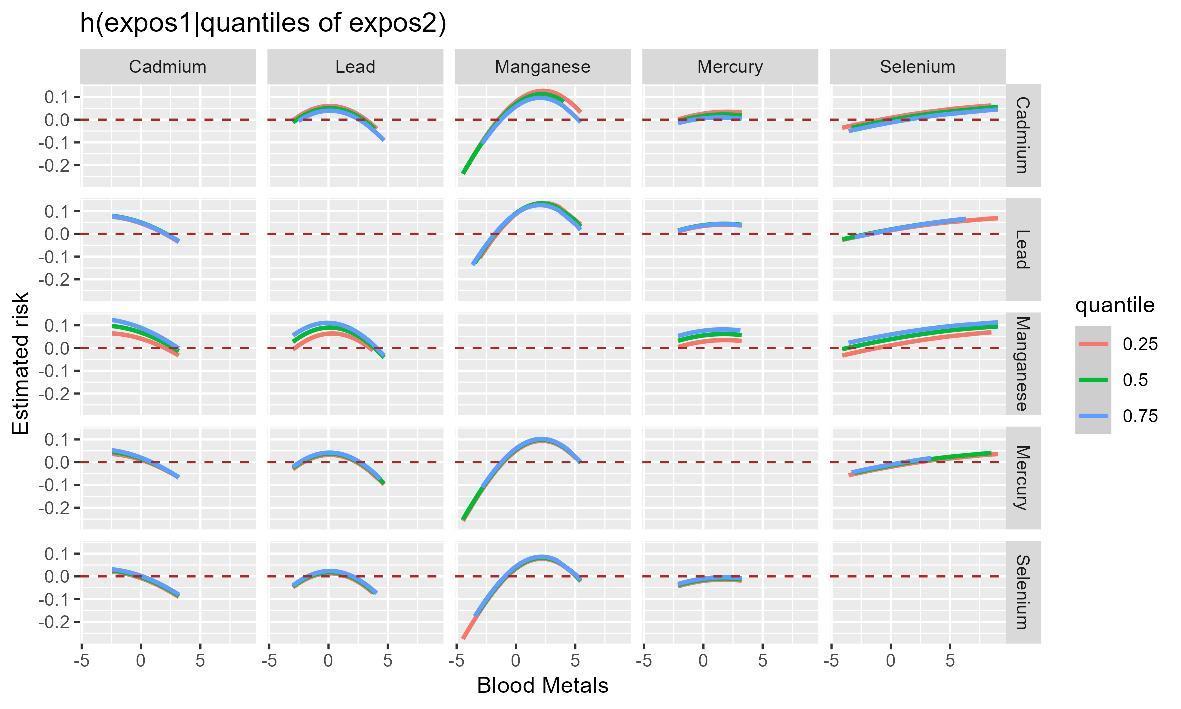


Fig S7 Bivariate exposure-response relationship for each trace element on DRT when the other trace elements were fixed at 25th, 50th, and 75th in the whole population by the BKMR model. The model was adjusted for adjusted by sex, age, race, education, income, marital status, physical activity, drinking, smoking, BMI, diabetes, hypertension, hyperlipidemia, and DII.


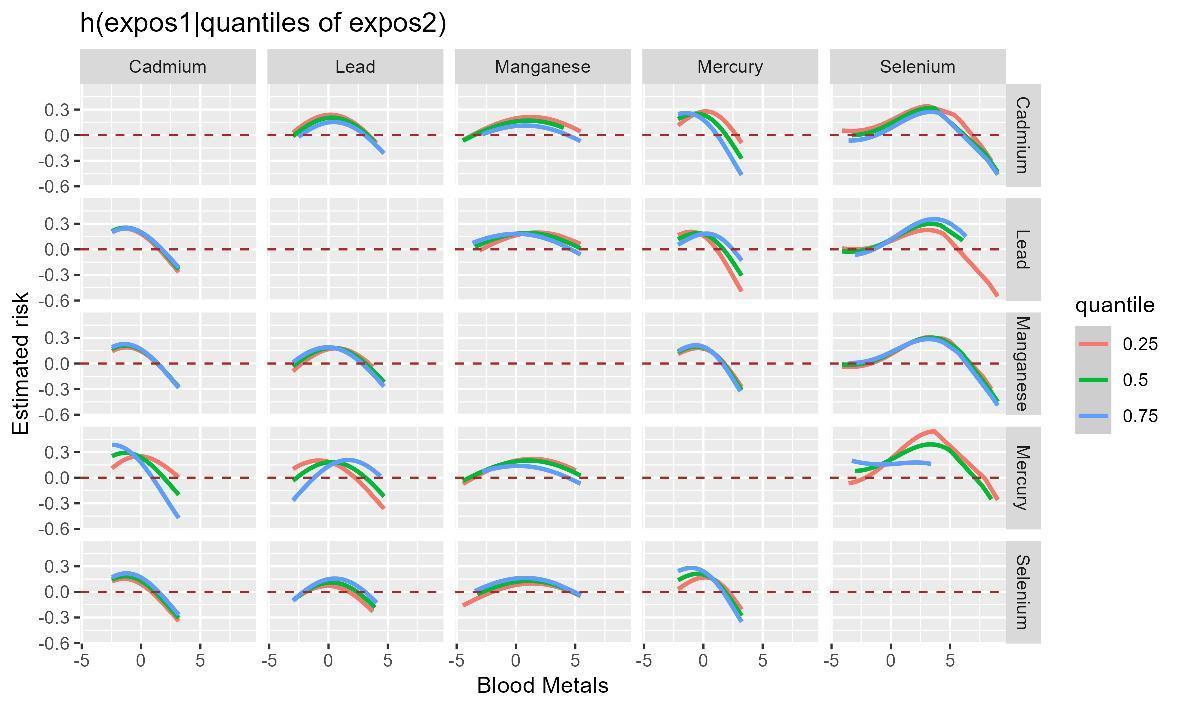


Fig S8 Bivariate exposure-response relationship for each trace element on AFT when the other trace elements were fixed at 25th, 50th, and 75th in the whole population by the BKMR model. The model was adjusted for adjusted by sex, age, race, education, income, marital status, physical activity, drinking, smoking, BMI, diabetes, hypertension, hyperlipidemia, and DII.


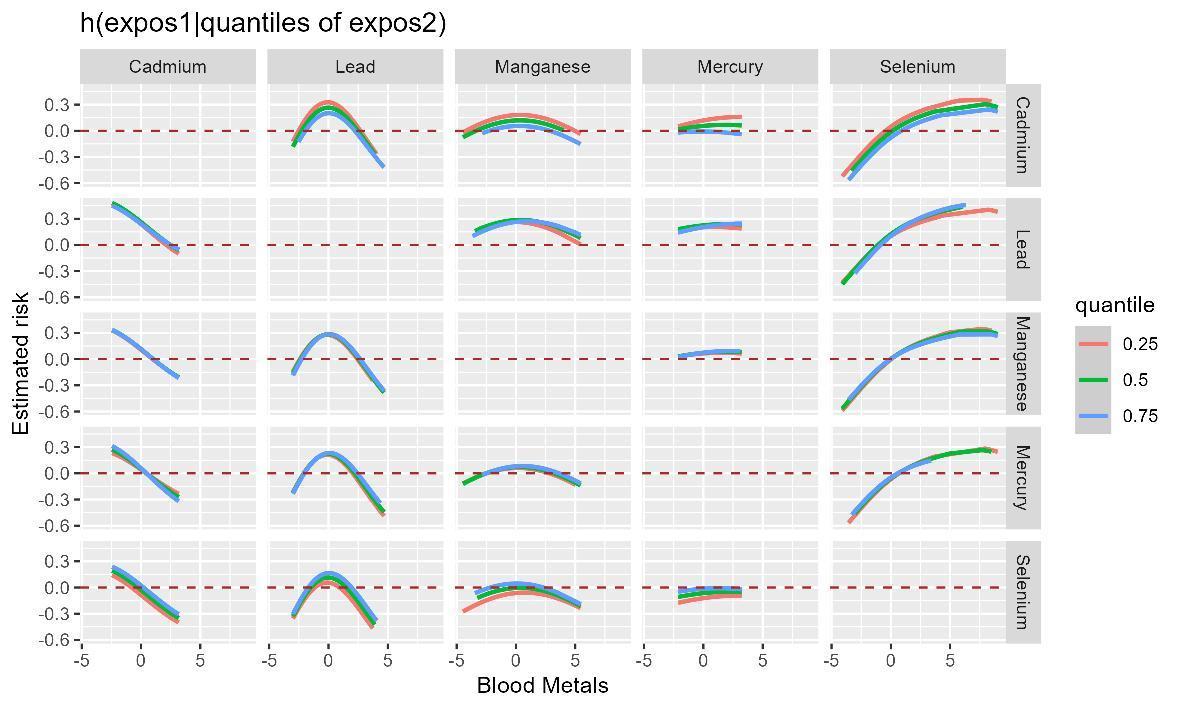


Fig S9 Bivariate exposure-response relationship for each trace element on DSST when the other trace elements were fixed at 25th, 50th, and 75th in the whole population by the BKMR model. The model was adjusted for adjusted by sex, age, race, education, income, marital status, physical activity, drinking, smoking, BMI, diabetes, hypertension, hyperlipidemia, and DII.


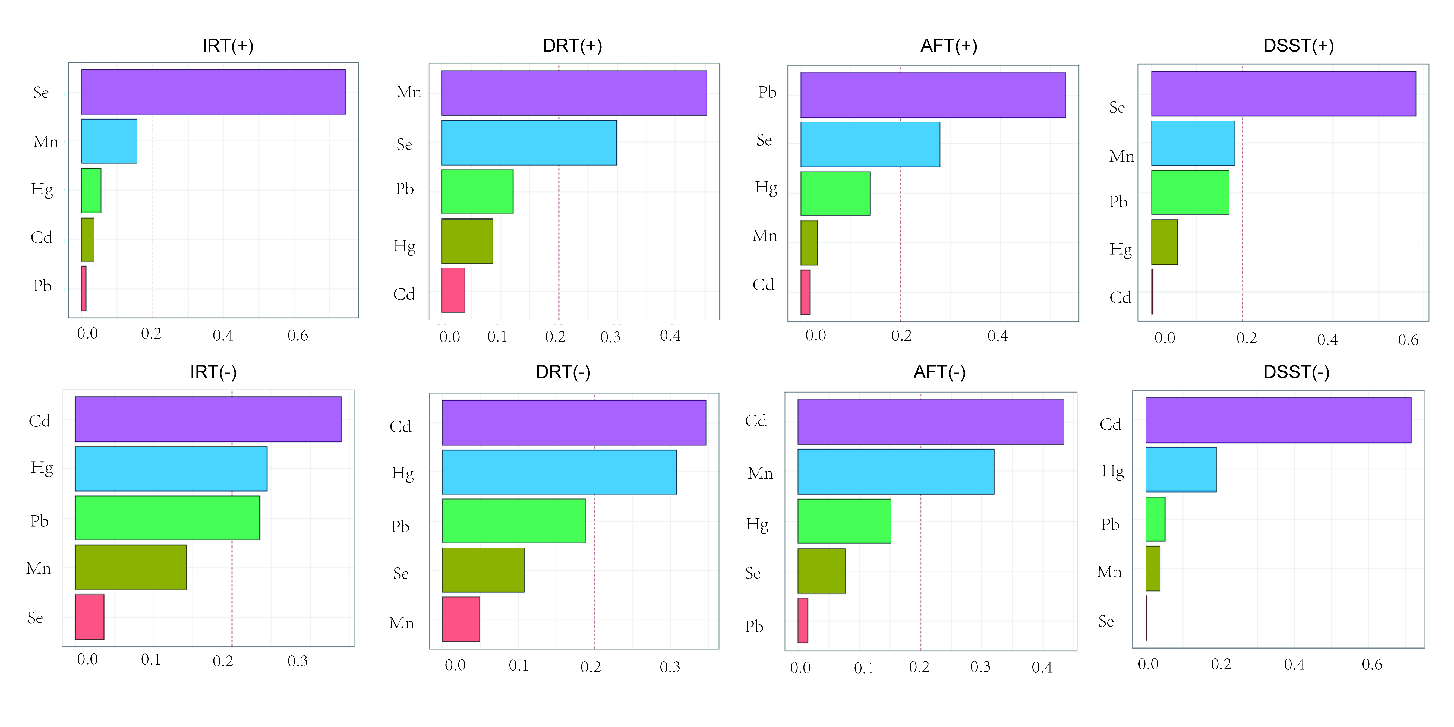


Fig S10 WQS model regression index weights for blood trace elements and cognitive function in the whole population. The model was adjusted for sex, age, race, education, income, marital status, physical activity, drinking, smoking, BMI, diabetes, hypertension, hyperlipidemia, and DII.


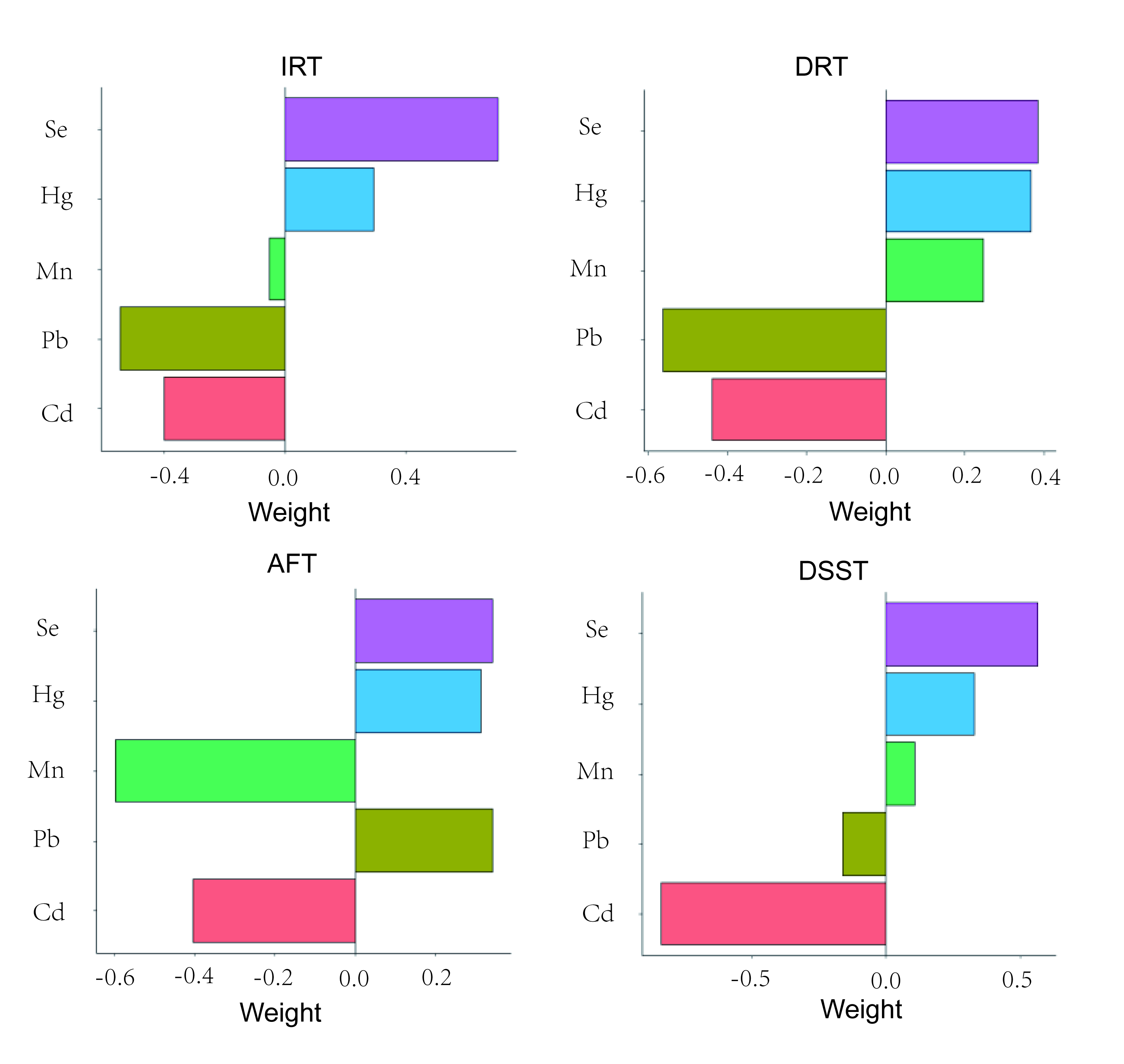


Fig S11 Qg-comp model regression index weights for blood trace elements and cognitive function in the whole population. The model was adjusted for sex, age, race, education, income, marital status, physical activity, drinking, smoking, BMI, diabetes, hypertension, hyperlipidemia, and DII.


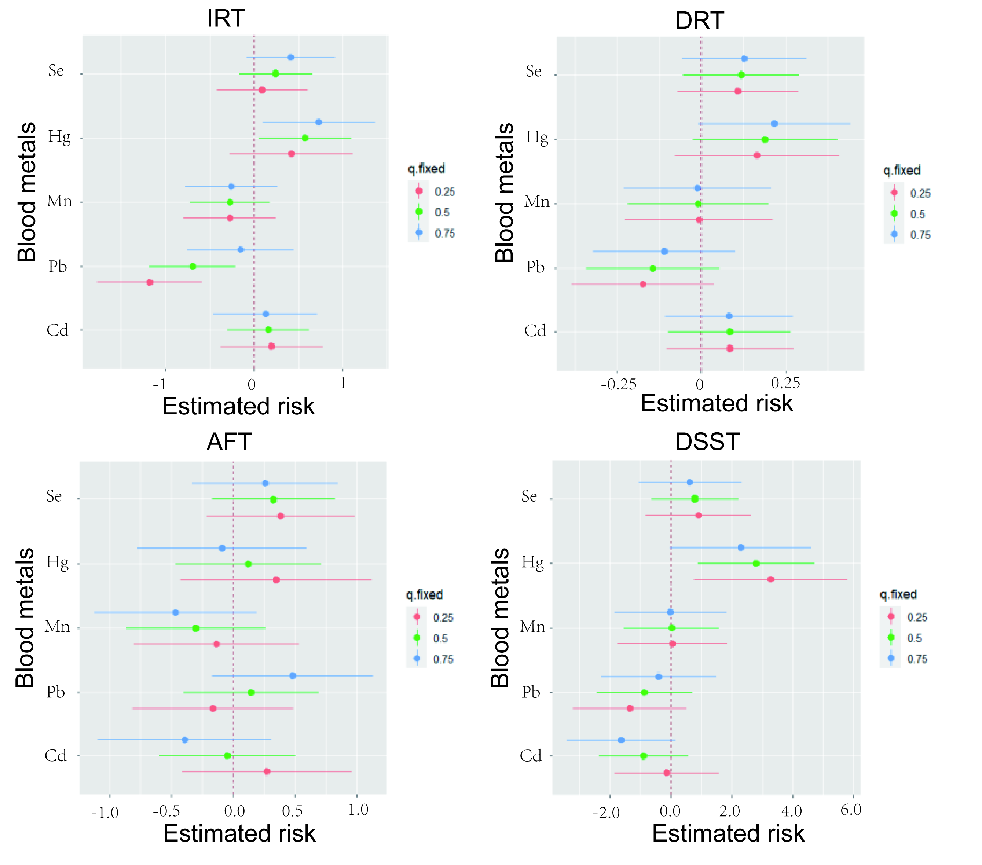


Fig S12 The effect of a single trace element variable at the 25th to 75th percentile on the IRT, DRT, AFT, and DSST by BKMR model analysis in the anti-inflammatory population when other trace elements were fixed at the 25th, 50th, and 75th percentiles.


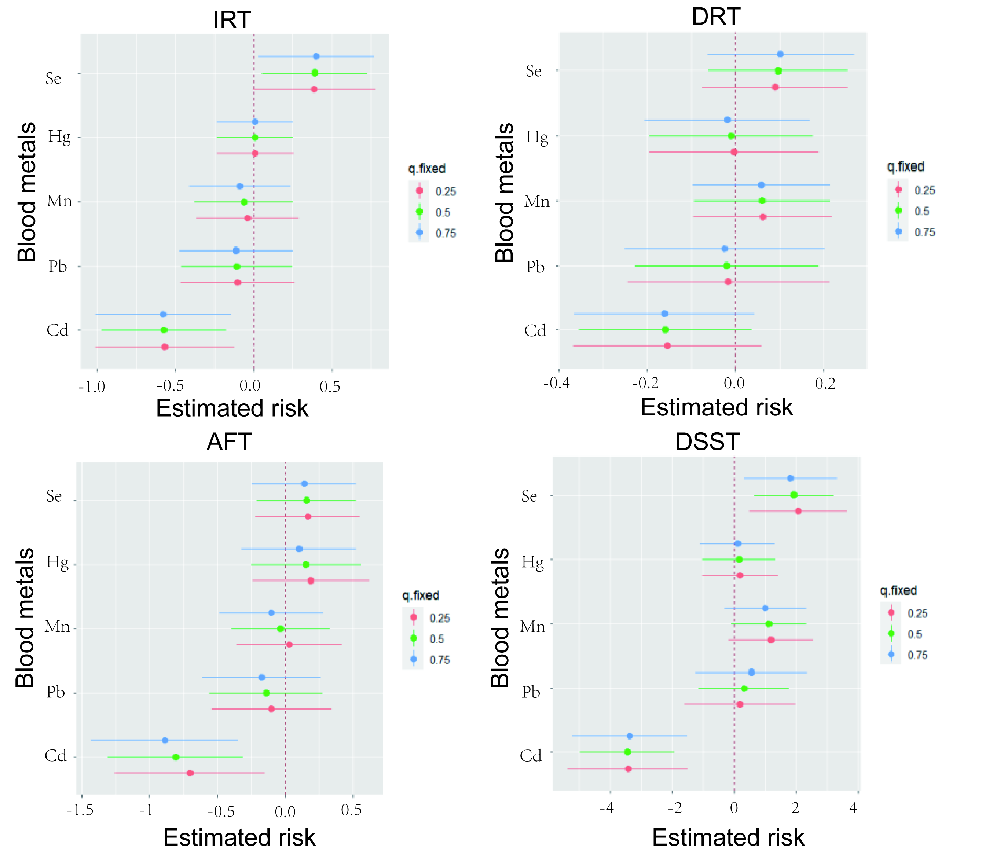


Fig S13 The effect of a single trace element variable at the 25th to 75th percentile on the IRT, DRT, AFT, and DSST by BKMR model analysis in the pro-inflammatory population when other trace elements were fixed at the 25th, 50th, and 75th percentiles.


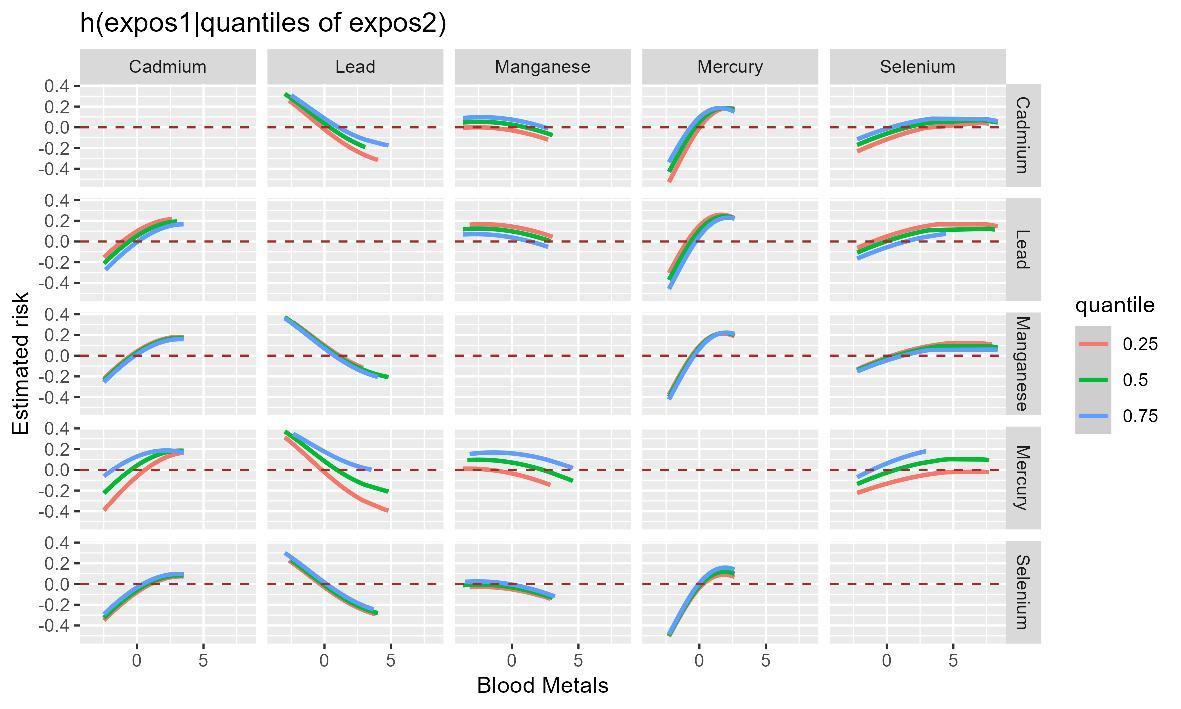


Fig S14 Bivariate exposure-response relationship for each trace element on IRT when the other trace elements were fixed at 25th, 50th, and 75th in the anti-inflammatory population by the BKMR model. The model was adjusted for adjusted by sex, age, race, education, income, marital status, physical activity, drinking, smoking, BMI, diabetes, hypertension, and hyperlipidemia.


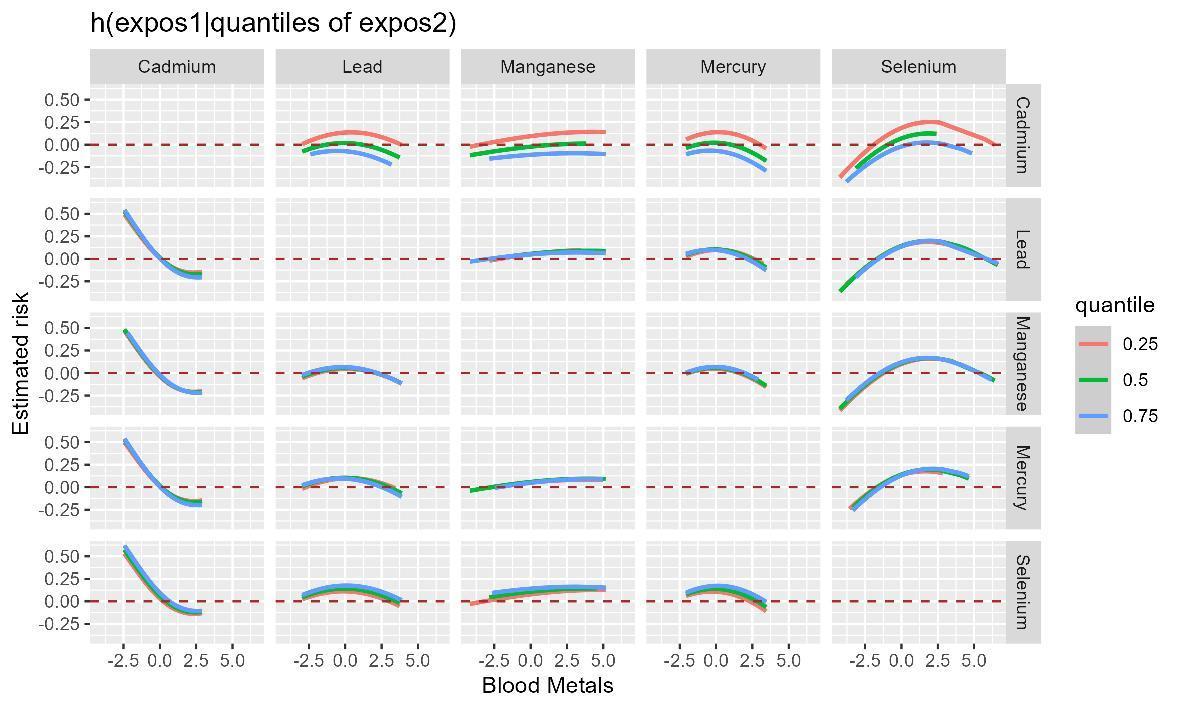


Fig S15 Bivariate exposure-response relationship for each trace element on DRT when the other trace elements were fixed at 25th, 50th, and 75th in the anti-inflammatory population by the BKMR model. The model was adjusted for adjusted by sex, age, race, education, income, marital status, physical activity, drinking, smoking, BMI, diabetes, hypertension, and hyperlipidemia.


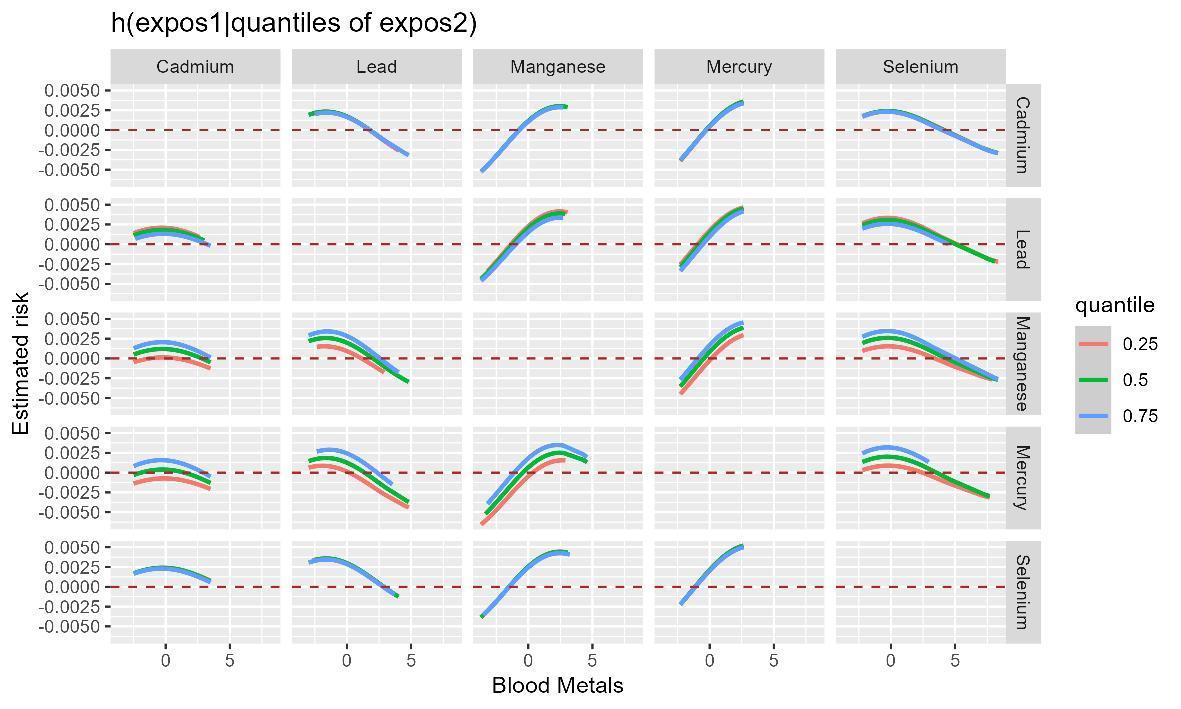


Fig S16 Bivariate exposure-response relationship for each trace element on AFT when the other trace elements were fixed at 25th, 50th, and 75th in the anti-inflammatory population by the BKMR model. The model was adjusted for adjusted by sex, age, race, education, income, marital status, physical activity, drinking, smoking, BMI, diabetes, hypertension, and hyperlipidemia.


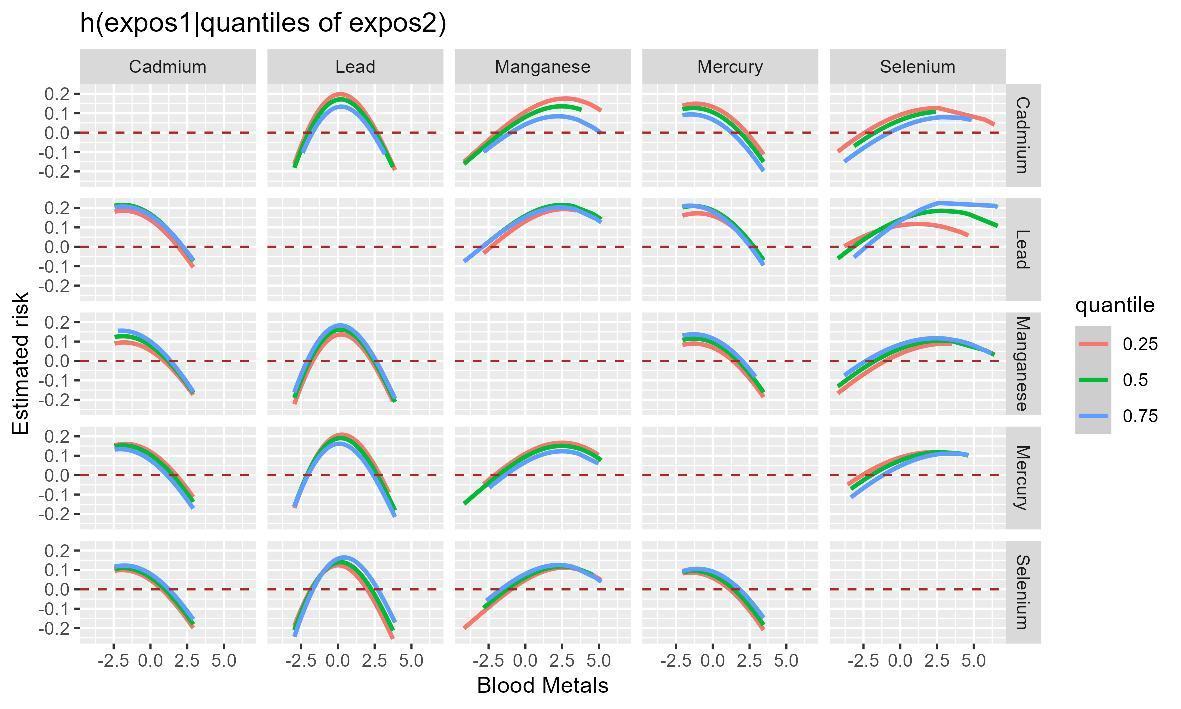


Fig S17 Bivariate exposure-response relationship for each trace element on DSST when the other trace elements were fixed at 25th, 50th, and 75th in the anti-inflammatory population by the BKMR model. The model was adjusted for adjusted by sex, age, race, education, income, marital status, physical activity, drinking, smoking, BMI, diabetes, hypertension, and hyperlipidemia.


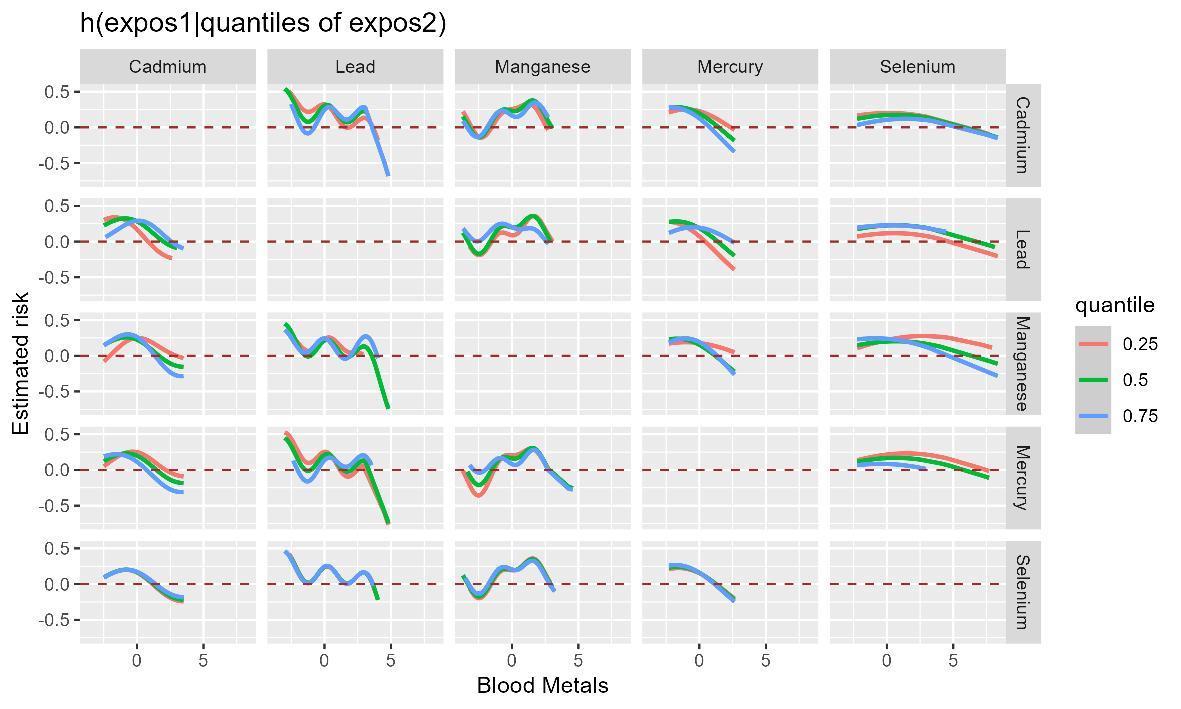


Fig S18 Bivariate exposure-response relationship for each trace element on IRT when the other trace elements were fixed at 25th, 50th, and 75th in the pro-inflammatory population by the BKMR model. The model was adjusted for adjusted by sex, age, race, education, income, marital status, physical activity, drinking, smoking, BMI, diabetes, hypertension, and hyperlipidemia.


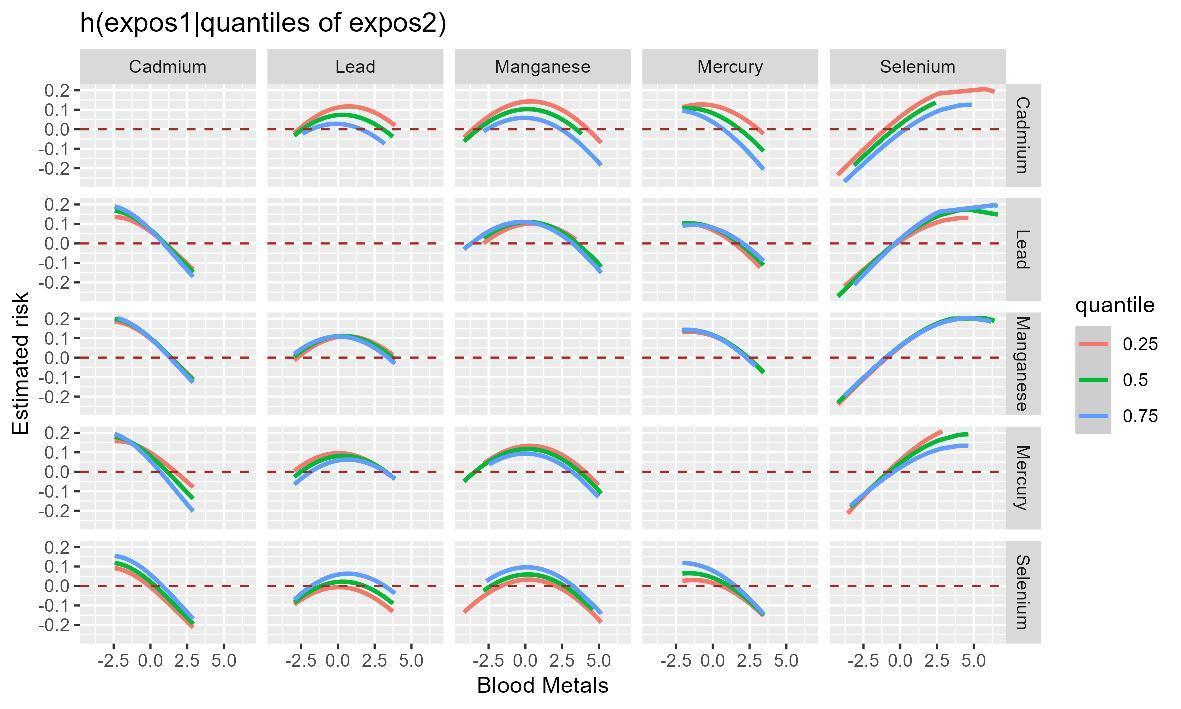


Fig S19 Bivariate exposure-response relationship for each trace element on DRT when the other trace elements were fixed at 25th, 50th, and 75th in the pro-inflammatory population by the BKMR model. The model was adjusted for adjusted by sex, age, race, education, income, marital status, physical activity, drinking, smoking, BMI, diabetes, hypertension, and hyperlipidemia.


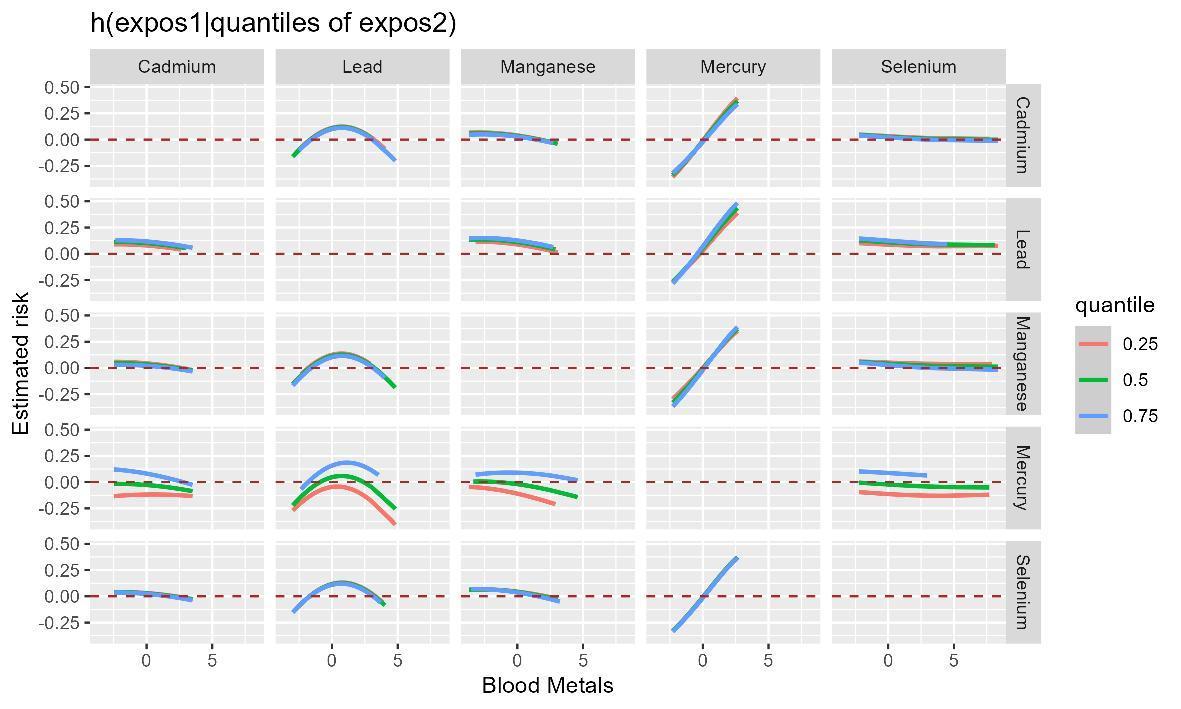


Fig S20 Bivariate exposure-response relationship for each trace element on AFT when the other trace elements were fixed at 25th, 50th, and 75th in the pro-inflammatory population by the BKMR model. The model was adjusted for adjusted by sex, age, race, education, income, marital status, physical activity, drinking, smoking, BMI, diabetes, hypertension, and hyperlipidemia.


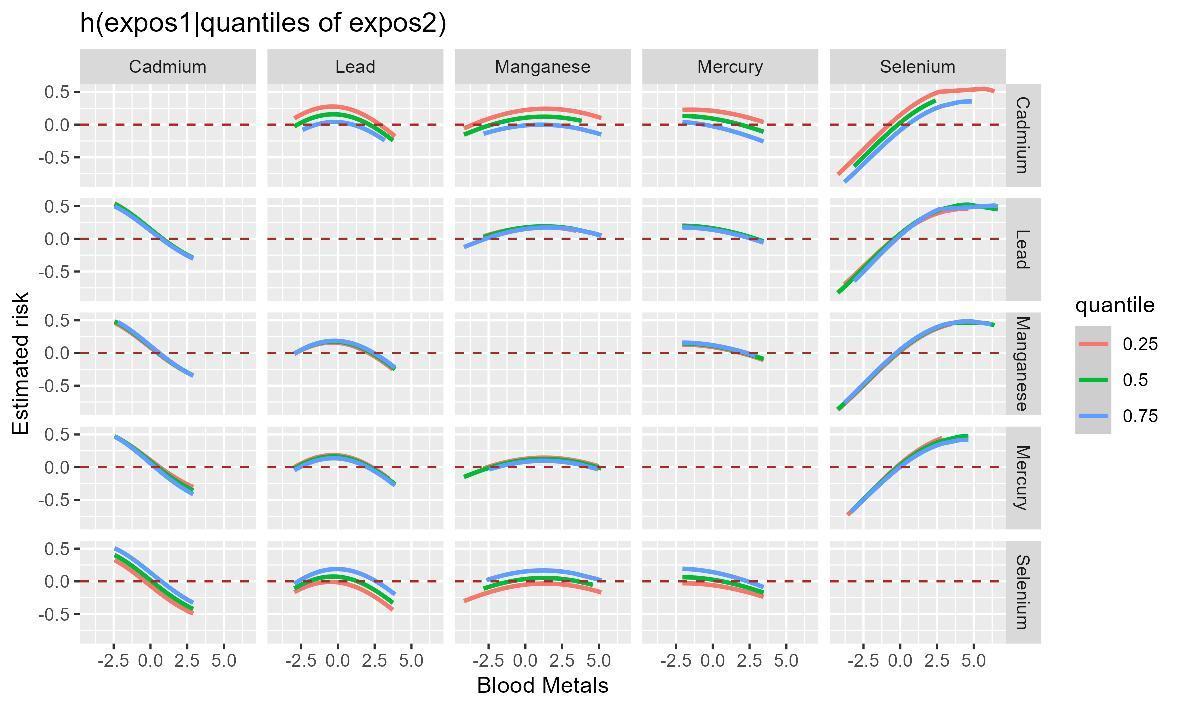


Fig S21 Bivariate exposure-response relationship for each trace element on DSST when the other trace elements were fixed at 25th, 50th, and 75th in the pro-inflammatory population by the BKMR model. The model was adjusted for adjusted by sex, age, race, education, income, marital status, physical activity, drinking, smoking, BMI, diabetes, hypertension, and hyperlipidemia.
